# Monocytes give rise to Langerhans cells that preferentially migrate to lymph nodes at steady state

**DOI:** 10.1073/pnas.2404927121

**Published:** 2024-11-14

**Authors:** Hayley M. Raquer-McKay, Raul A. Maqueda-Alfaro, Sanjana Saravanan, Rebeca Arroyo Hornero, Björn E. Clausen, Andres Gottfried-Blackmore, Juliana Idoyaga

**Affiliations:** ^a^Microbiology and Immunology Department, Stanford University School of Medicine, Stanford, CA 94305; ^b^Immunology Program, Stanford University School of Medicine, Stanford, CA 94304; ^c^Pharmacology Department, School of Medicine, University of California San Diego, La Jolla, CA 92093; ^d^Institute for Molecular Medicine, Paul Klein Center for Immune Intervention, University Medical Center of the Johannes Gutenberg-University Mainz, Mainz 55131, Germany; ^e^Research Center for Immunotherapy (Forschungs-Zentrum für Immuntherapie), University Medical Center of the Johannes Gutenberg-University Mainz, Mainz 55131, Germany; ^f^Department of Medicine, Division of Gastroenterology, University of California San Diego, La Jolla, CA 92093; ^g^Veterans Affairs San Diego Healthcare System, Gastroenterology Section, La Jolla, CA 92161; ^h^Molecular Biology Department, School of Biological Sciences, University of California San Diego, La Jolla, CA 92093

**Keywords:** macrophages, development, skin, migration, Langerhans cells

## Abstract

Current evidence suggests that ontogeny may account for the functional heterogeneity of some tissue macrophages, but not others. Here, we asked whether developmental origin drives different functions of skin Langerhans cells (LCs), an embryo-derived mononuclear phagocyte with features of both tissue macrophages and dendritic cells. Using time-course analyses, bone marrow chimeras, and fate tracing models, we found that the complete elimination of embryo-derived LCs at steady state results in their repopulation from circulating monocytes. However, monocyte-derived LCs inefficiently replenished the epidermal niche. Instead, these cells preferentially migrated to skin-draining lymph nodes. Mechanistically, we show that the enhanced migratory capability of monocyte-derived LCs is associated with higher expression of CD207/Langerin, a C-type lectin involved in the capture of skin microbes. Our data demonstrate that ontogeny plays a role in the migratory behavior of epidermal LCs.

## Significance

Several tissues, such as the skin, are populated with macrophages derived from embryonic precursors. These tissue macrophages scavenge debris and microbes to maintain organ function. Depletion of embryo-derived tissue macrophages can result in the recruitment of monocyte-derived macrophages that occupy the emptied tissue. Understanding how macrophage origin regulates their tissue functions is an unmet need for the design of therapeutics. This study compares embryo-derived and monocyte-derived Langerhans cells (LCs), macrophage-related cells localized in the epidermis of humans and mice. We show that monocyte-derived LCs have a superior migratory capacity than embryo-derived LCs due to their distinct expression of receptors for the uptake of microbes. Our findings shed light on LC heterogeneity and open venues for harnessing these cells therapeutically.

Macrophages are integral components of all organs, playing distinct roles in maintaining tissue-specific and immune scavenging functions (1–3). These mononuclear phagocytes develop prenatally from embryonic progenitors and are either maintained through adulthood by self-renewal or are replaced partially or totally by circulating monocytes (4–10). The contribution of embryonic progenitors and circulating monocytes to the homeostatic pool of adult macrophages has been mapped and is known to be tissue-specific (11, 12). However, it is still debated whether ontogeny plays a role in modulating macrophage functions within the same tissue. Tissue-specific factors, as opposed to ontogeny, determine gene expression and function for lung and liver macrophages (13–17). Contrarily, the morphology and gene expression of brain microglia seem dependent on their origin (18–20). These discrepancies suggest that the contribution of ontogeny to the functional heterogeneity of macrophages is also tissue dependent and highlights the need to correlate origin and functional heterogeneity for each population of tissue macrophages.

The skin epidermis provides the first barrier of protection from the invasion of pathogens into the body. In humans and mice, the epidermis is populated by a distinct population of mononuclear phagocytes, i.e., Langerhans cells (LCs). LCs arise from embryonic progenitors that seed the skin before birth (21–24) and consequently are considered macrophages with regard to their origin. Similar to other embryo-derived macrophages, LCs are long lived and are thought to be locally sustained in the adult epidermis through low levels of proliferation (25–27). However, different from other tissue macrophages, LCs migrate to skin-draining lymph nodes (sLN) at steady state and instruct the generation of regulatory T cells (28–34), a function usually associated with dendritic cells (DCs). This is the reason why LCs were sensibly called “*The macrophage in dendritic cell clothing*” (35). Importantly, the one-way migration of LCs to sLN poses a distinct challenge for the maintenance of the epidermal niche. Until now, it is still unclear whether slow self-renewal during homeostasis is the only mechanism that sustains LC numbers in the epidermis and sLN or whether there is heterogeneity within LC that differentially maintains these two niches. Recent work suggested the existence of LC subpopulations with differential migratory capabilities (36); however, a correlation between the origin of these homeostatic LC subpopulations and their migration capabilities was not systematically analyzed. Importantly, the presence of LC subpopulations with distinct ontogeny and migration capabilities may help explain the dual inflammatory and anti-inflammatory functions attributed to these cells (31, 37–41).

Notably, two distinct LC subpopulations with different origins, i.e., embryonic and monocytic, can be found during skin inflammation (42, 43). Originally, it was proposed that inflammation promotes the recruitment of monocytes and their differentiation to LCs that survey the skin for short periods of time (43). These “short-term” LCs were thought to be replaced by long-term LCs derived from undefined progenitors. However, with the advent of new technologies, Ferrer et al. recently showed that monocyte-derived LCs (moLCs) seed the skin and maintain themselves by self-renewal for a prolonged period of time during graft-versus-host disease (GVHD) (44). In this inflammatory setting, moLCs are transcriptionally indistinct from embryo-derived LCs (eLCs) (44), suggesting that the skin environment, rather than ontogeny, is the key factor contributing to function. Whether this is also true at steady state for LCs localized in the epidermis and sLN has not been evaluated yet.

Here, we leveraged a genetic mouse model of noninflammatory eLC depletion that allowed us to characterize the replenishment of both niches, the epidermis and sLN, in parallel at steady state. We found that similar to the inflammatory setting, depletion of eLCs results in repopulation with moLCs. However, at steady state, moLCs repopulated the epidermis poorly and were mainly found in the sLN niche. Competitive analysis demonstrated that moLCs have an intrinsic superior capacity to migrate to sLN than eLCs. Mechanistically, we show that moLCs express higher levels of the C-type lectin CD207/Langerin that can mediate migration through the capture of skin microbes. Our data support the concept that ontogeny regulates a key function of LCs at steady state.

## Results

### LCs Repopulate the Skin-Draining Lymph Nodes, but Not the Epidermis, Following Their Depletion at Steady State.

To investigate the dynamics of LC repopulation in the skin and sLN in parallel, we depleted these two niches and followed the process of reconstitution over time, as previously done for other tissue macrophages (17, 45). CD207/Langerin is a C-type lectin expressed by epidermal LCs and by dermal DC type 1 (DC1s) (46–48). CD207 is also expressed by all lymphoid-resident DC1 localized in sLN of Balb/c mice (49). Thus, Balb/c *Cd207*^DTR^ mice allowed us to compare LC repopulation with that of dermal and lymphoid-resident DC1s. A single dose of DT resulted in the complete elimination of all CD207-expressing cells in the skin and sLN by 48 h, without depleting other mononuclear phagocytes (*SI Appendix*, Fig. S1 *A*, *B*, *D*, and *E*). DT did not trigger the recruitment of neutrophils or eosinophils, suggesting that it does not result in overt inflammation (*SI Appendix*, Fig. S1*C*).

**Fig. 1.**
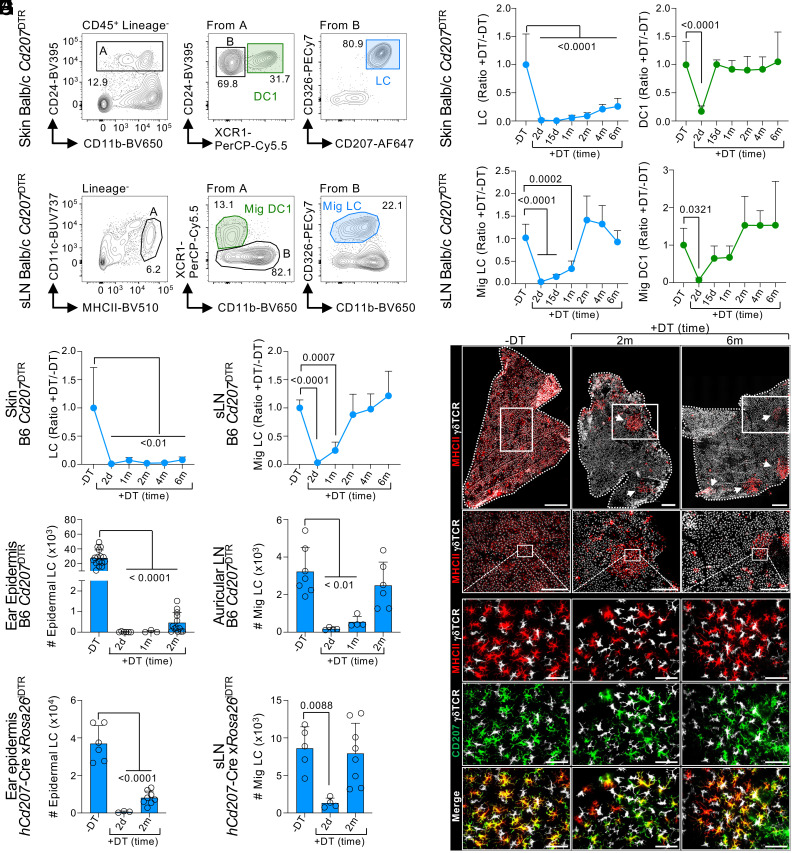
LCs repopulate sLN, but not the epidermis, after their depletion at steady state. (*A*–*D*) Balb/c *Cd207*^DTR^ mice were inoculated or not with 50 ng/gr body weight DT. Ears and sLN (popliteal, inguinal, brachial, and axillary) were harvested, and cell suspensions were stained for flow cytometry analysis. (*A*) Gating strategy to identify skin LCs and DC1s. (*B*) Skin LC (*Left*) and DC1 (*Right*) numbers were quantified over time and normalized to non-DT controls. Shown is the mean + SD (n = 5-13 mice in 4 to 10 exp.; one-way ANOVA with Dunnett’s multiple comparison). (*C*) Gating strategy to identify migratory LCs (Mig LC) and DC1s (Mig DC1) in sLN. (*D*) Mig LC (*Left*) and Mig DC1 (*Right*) numbers were quantified and normalized to non-DT controls (n = 4-19 mice in 4 to 10 exp.; one-way ANOVA with Dunnett’s multiple comparison). (*E*–*G*) B6 *Cd207*^DTR^ mice were treated or not with 50 ng/gr body weight DT. (*E*) LCs were quantified in the skin (*Left*) and sLN (*Right*) and normalized to non-DT controls (n = 3-22 mice in 2 to 7 exp.; one-way ANOVA with Dunnett’s multiple comparison). (*F*) The ear epidermis was stained for MHCII^+^CD207^+^ LCs and γ δTCR^+^ T cells by microscopy (1 of 4 exp.). *Top* panel, epidermal sheet stitching (scale bar: 500 μm); second row, magnification of white squares from the *Top* panel (scale bar: 100 μm); and *Bottom* panels, magnification of second row (scale bar: 50 μm). (*G*) LC numbers were quantified in the ear epidermis (*Left*; n = 3-12 mice in 2 to 4 exp.; one-way ANOVA with Dunnett’s multiple comparison) and ear-draining auricular LN (n = 4-7 mice in 2 to 3 exp.; one-way ANOVA with Dunnett’s multiple comparison). (*H*) *hCd207*-Cre x*Rosa26*^iDTR^ mice were inoculated or not with 50 ng/gr body weight DT. Numbers of epidermal LCs (*Left*) and Mig LCs in sLN (*Right*) are shown as the mean + SD (n = 3-8 mice in 3 exp.; one-way ANOVA with Dunnett’s multiple comparison). d: days; m: months; Mig: migratory.

Time-course analyses of the skin showed that dermal DC1s returned to normal numbers 15 d post-DT inoculation (Fig. 1 *A* and *B*). However, skin LC numbers remained significantly lower, even after 6 mo post-DT inoculation. The low LC numbers post-DT were observed in both our whole-skin and epidermal cell preparations (*SI Appendix*, Fig. S2 *A* and *B*), implying that it was not a consequence of different skin digestion protocols. Parallel analysis of sLN showed that lymphoid-resident DC1 repopulated the sLN niche by 15 d post-DT inoculation (*SI Appendix*, Fig. S2*C*). Similarly, migratory DC1 in sLN returned to control levels by 15 d post-DT (Fig. 1 *C* and *D*), in accordance with their replenishment in the skin. Strikingly, despite the low number of skin LCs at 2 to 6 mo postdepletion, migratory LCs completely repopulated the sLN by 2 mo (Fig. 1*D*). This disconnect between skin and sLN LCs was also observed in B6 *Cd207*^DTR^ mice (Fig. 1*E*), thus being independent of the mouse background.

To confirm that LCs were unable to repopulate the skin, we performed several experiments. First, epidermal sheets were analyzed by microscopy (Fig. 1*F*). By 2 mo post-DT, only a few small patches of MHCII^+^ CD207^+^ LCs were detected, as described (37, 50). Even at 6 mo post-DT, LC patches did not achieve the density observed in non-DT controls. Second, the ear and back epidermis of B6 *Cd207*^DTR^ mice were compared to assess for anatomic-specific LC dynamics (Fig. 1*G* and *SI Appendix*, Fig. S2*D*). LCs were unable to repopulate either site at 2 mo. However, migratory LCs repopulated the ear and back skin-draining lymph nodes equally well. Third, we confirmed LC repopulation dynamics in a mouse model that specifically depletes LCs and does not affect DC1s, i.e., human *Cd207*-Cre mouse crossed to *Rosa26*^iDTR^ (51). In this model, LCs did not refill the epidermal niche after their depletion, but their numbers in sLN were similar to non-DT controls (Fig. 1*H*). Finally, we observed similar results using a model that does not require CD207 expression for LC depletion, i.e., *LysM*^Cre^ × *Csf1r*^LSL-DTR^ (*SI Appendix*, Fig. S2*E*). The disconnect between epidermal and sLN repopulation was only observed at steady state since LCs quickly repopulated the epidermis if inflammation was induced by tape stripping (*SI Appendix*, Fig. S2*F*). Notably, LCs found in the dermis, on their migratory path to sLN, followed the same kinetic of reconstitution as LCs in sLN, i.e., their numbers returned to normal levels by 2 mo (*SI Appendix*, Fig. S2*G*). Our results show that LC repopulation at steady state favors the sLN rather than the epidermis.

### Depleted Embryo-Derived LCs Are Replaced by Monocyte-Derived LCs.

**Fig. 2.**
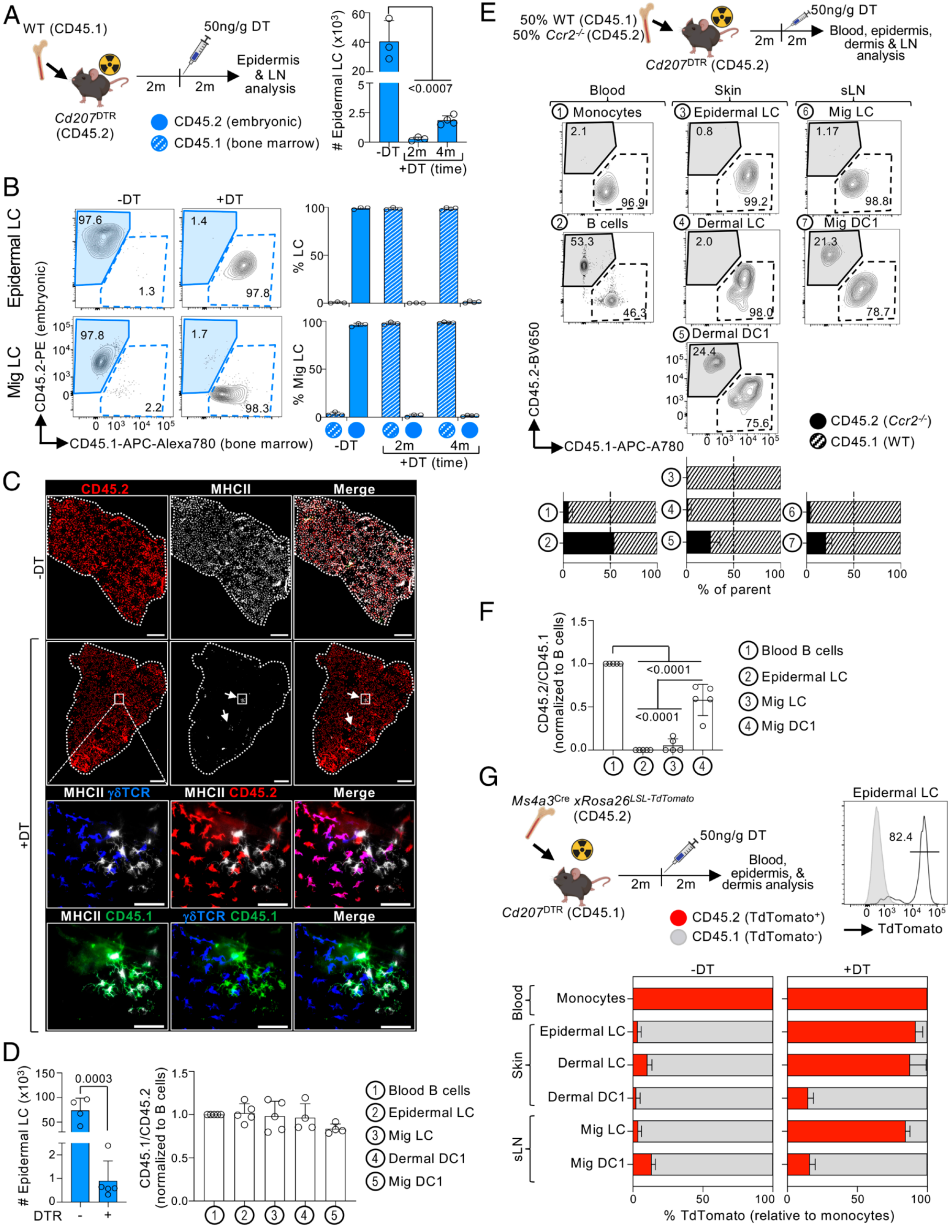
Monocytes give rise to LCs at steady state. (*A*–*C*) CD45.2 B6 *Cd207*^DTR^ mice were lethally irradiated and transplanted with CD45.1 WT bone marrow, followed by inoculation with 50 ng/gr body weight of DT. The epidermis and sLN were analyzed at 2 or 4 mo post-DT. (*A*) Experimental design (*Left*) and epidermal LC numbers as the mean + SD (n = 3-4 mice in 2 to 3 exp.; one-way ANOVA with Dunnett’s multiple comparison). (*B*) Expression of CD45.2 and CD45.1 in epidermal LCs (*Top*) and Mig LCs (*Bottom*). Representative flow cytometry analysis (*Left*) and mean + SD (n = 3-4 mice in 2 to 3 exp.; *Right*). (*C*) The ear epidermis was stained for MHCII^+^ LCs, radioresistant γδTCR^+^ T cells, CD45.1, and CD45.2 by microscopy 4 mo post-DT (1 of 3 exp.). *Top* panels, epidermal sheet stitching (scale bar: 500 μm); *Bottom* panels, magnified region of white squares (scale bar: 50 μm). (*D*) As in (*A*), but the ears and shoulders of *Cd207*^WT^ (DTR-) and *Cd207*^DTR^ (DTR+) mice were shielded before 9 Gy irradiation and bone marrow transplantation, followed by inoculation with 50 ng/gr body weight of DT. *Left* shows epidermal LC numbers (mean + SD; Student’s *t* test) at 2 mo post-DT. *Right* shows the mean + SD of CD45.1/CD45.2 expression normalized to blood B cells (n = 4-5 mice in 3 exp.; one-way ANOVA with Dunnett’s multiple comparison). (*E*) B6 CD45.2 *Cd207*^DTR^ mice were lethally irradiated and transplanted with 50% *Ccr2^−/−^*(CD45.2) and 50% WT (CD45.1) bone marrow, followed by 50 ng/gr body weight DT. *Top* shows experimental design. *Bottom* shows a representative flow cytometry staining, and the mean + SD of CD45.1/CD45.2 expression 2 mo post-DT (n = 3 mice in 1 exp.). (*F*) As in (*E*), but the ears and shoulders of mice were shielded before irradiation (9 Gy) and transplantation with 50% *Ccr2^−/−^*(CD45.2) and 50% WT (CD45.1) bone marrow. Shown is the mean + SD of CD45.2/CD45.1 expression normalized to blood B cells (n = 5 mice in 1 exp; one-way ANOVA with Tukey’s multiple comparison). (*G*) B6 CD45.1 *Cd207*^DTR^ mice were lethally irradiated, transplanted with CD45.2 *Ms4a3*^cre^ x*Rosa26*^LSL-TdTomato^ bone marrow, and inoculated with 50 ng/gr body weight DT. *Top* shows the experimental design and a representative flow cytometry analysis of TdTomato expression by epidermal LCs 2 mo post-DT. *Bottom* shows the mean + SD of TdTomato expression (n = 4-7 mice in 3 exp.). m: months.

To evaluate the origin of the repopulating LCs, we took advantage of the radioresistant feature of embryonic LCs to generate congenic bone marrow chimeras (BMC) by transplanting CD45.1 wild-type (WT) bone marrow into lethally irradiated CD45.2 *Cd207*^DTR^ mice (27). Two months post-transplantation, embryonic LCs were depleted by DT inoculation, and the origin (host or bone marrow) of returning LCs was followed over time. Similar to our previous observations, returning LCs were unable to repopulate the epidermis by 2- and 4-mo post-DT (Fig. 2*A*). LCs from non-DT-inoculated mice had an embryonic origin, as expected (Fig. 2*B*). However, the few LCs found in the skin 2- and 4-mo post-DT were all derived from bone marrow progenitors. Matching their epidermal counterpart, repopulating migratory LCs in sLN were also derived from bone marrow progenitors (Fig. 2*B*). The bone marrow origin and epidermal localization of repopulating LCs were confirmed by microscopy analysis. Again, a few patches of returning LCs were found, but all had a bone marrow CD45.1^+^ origin (Fig. 2*C*). To confirm that LC repopulation with bone marrow–derived progenitors was not a result of low-grade inflammation induced by irradiation, we generated shielded BMC as previously described for other tissue macrophages (6, 17, 20). The head and shoulders of CD45.2 *Cd207*^DTR^ mice were shielded from radiation to prevent damage in the ear skin and sLN before engraftment with CD45.1 WT bone marrow, followed by DT inoculation. Skin and migratory LCs of head-and-shoulder-shielded BMC showed a chimerism that matched blood B cells (Fig. 2*D*), suggesting that the bone marrow origin of repopulating LCs is unlikely to be a confounding effect of irradiation.

During skin inflammation, bone marrow monocytes differentiate into LCs (42–44). To determine whether monocytes also mediate homeostatic LC repopulation, we generated a competitive BMC by lethally irradiating *Cd207*^DTR^ mice and transplanting them with 50% WT and 50% *Ccr2*^−/−^ bone marrow, as CCR2 is a chemokine receptor necessary for monocyte egress from the bone marrow (52). Analysis of blood showed that WT outcompeted *Ccr2*^−/−^ bone marrow for the reconstitution of monocytes, but not B cells (Fig. 2*E*). After DT-mediated elimination of embryonic LCs, WT but not *Ccr2*^−/−^ bone marrow contributed exclusively to the reconstitution of skin LCs. WT bone marrow also had a slightly larger contribution for DC1 reconstitution, in agreement with the recently reported role of CCR2 in DC progenitors (53). The contribution of WT and *Ccr2*^−/−^ bone marrow to migratory LCs and DC1s in sLN matched their skin counterparts. Similar results were observed when repeating these experiments with head-and-shoulder-shielded BMC (Fig. 2*F*), suggesting that these results are unlikely to be a consequence of irradiation-induced inflammation. Thus, LC repopulation at steady state requires CCR2-dependent bone marrow cells.

It has been suggested that DC progenitors could give rise to human LCs (54–56). To dissect the contribution of monocytes vs. DC progenitors to homeostatic LC repopulation, we transplanted lethally irradiated *Cd207*^DTR^ with *Ms4a3*^Cre^ × *Rosa26*^LSL-TdTomato^ bone marrow, that traces monocytes and monocyte-derived cells (57) (Fig. 2*G*). After DT inoculation, most DC1s were TdTomato^−^; however, the majority of the epidermal, dermal, and migratory LCs were TdTomato^+^, demonstrating their monocytic origin. Notably, inflammation induced by tape-stripping also resulted in monocyte-derived LC repopulation (*SI Appendix*, Fig. S3*A*).

Our results show that depletion of embryonic LCs (eLCs) at steady state results in repopulation with monocyte-derived LCs (moLCs). We next aimed at understanding the timing of monocyte infiltration to the skin after eLC depletion. Following eLC elimination, we were unable to detect any significant infiltration of monocytes at any time point analyzed (*SI Appendix*, Fig. S3 *B* and *C*), in contrast to other models of tissue macrophage depletion at steady state (17, 44, 45). We were also unable to detect an accumulation of MHCII^+^ CD11b^+^ cells, i.e., cells in their developmental transition to LCs (44). We hypothesized that a small number of infiltrating monocytes may quickly differentiate into moLCs that then undergo proliferation. Indeed, we observed that epidermal moLCs undergo a peak of proliferation measured by Ki67 expression at 2 mo post-DT inoculation (*SI Appendix*, Fig. S3*D*), the time point in which the sLN niche is replenished (Fig. 1*D*).

### sLN Monocyte-Derived LCs Are Coming from the Skin.

**Fig. 3.**
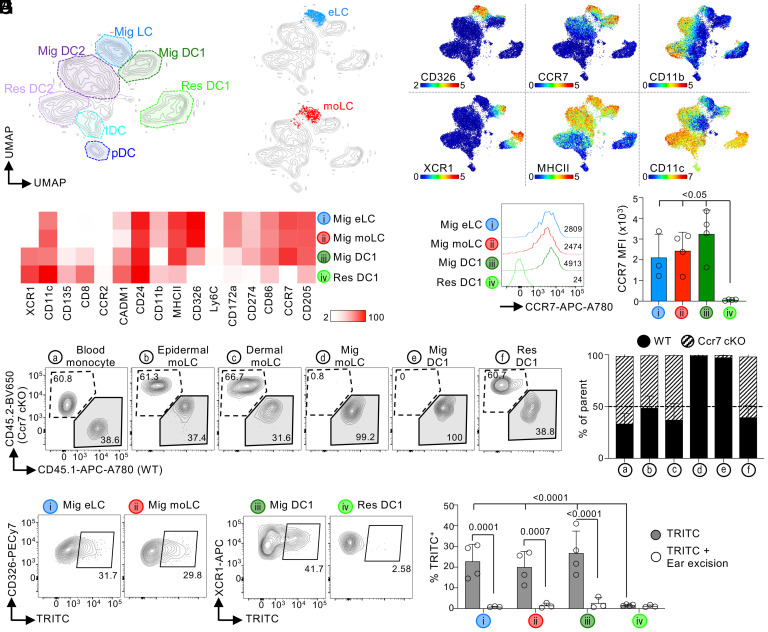
moLCs migrate from the skin to sLN. (*A*–*D*) B6 *Cd207*^DTR^ mice were inoculated or not with 50 ng/gr body weight DT. Two months later, Mig LCs were analyzed by CyTOF. (*A*) UMAP showing all myeloid cells in sLN (1 of 3 exp.). (*B*) eLCs (−DT; *Top*), and moLCs (+DT; *Bottom*) were overlaid into the UMAP of all sLN cells (1 of 3 exp.). (*C*) UMAPs showing the expression of selected markers (1 of 3 exp.; scale: ArcSinh). (*D*) Heatmap of the expression of each marker in each cell population (n = 3 mice in 3 exp.; scale: MSI). (*E*) CCR7 expression by flow cytometry (1 of 3 exp.). A representative flow cytometry plot (*Right*) and the mean fluorescence intensity (MFI) + SD (n = 3-4 mice in 3 exp.; *Left*). (*F* and *G*) B6 *Cd207*^DTR^ CD45.2 mice were lethally irradiated and transplanted with 50% WT (CD45.1) and 50% Ccr7 cKO bone marrow (CD45.2), followed by 50 ng/gr body weight DT. (*F*) Representative flow cytometry plots are shown. (*G*) As in (*F*), but the frequency of cells expressing CD45.1 or CD45.2 (mean + SD; n = 9 mice in 2 exp.). (*H*) B6 *Cd207*^DTR^ mice were treated or not with 50 ng/gr body weight of DT. Two months later, TRITC was applied onto the ears. In some cases, ears were excised 4 h after TRITC application. Auricular LN were collected 96 h later for flow cytometry analysis. *Left* shows representative plots. *Right* shows the frequency of TRITC^+^ cells (mean + SD; n = 3-4 mice in 2 exp.; two-way ANOVA with Fisher’s Least Significant Difference). Res: lymphoid-resident; MSI: Mean Signal Intensity.

A distinctive trait of LCs is their migration from the epidermis to the sLN. We asked whether similar to eLCs, moLCs localized in sLN have a phenotype of skin migratory cells using Cytometry by Time of Flight (CyTOF) (Fig. 1*D*). Unbiased clustering showed that sLN moLCs and eLCs fall within the same region of the UMAP (Fig. 3 *A* and *B*), demonstrating that they have a similar phenotype, including markers associated with DC migration from the skin such as CCR7, MHCII, and CD86 (Fig. 3 *C* and *D*).

The chemokine receptor CCR7 mediates skin DC migration to sLN (58). Flow cytometry quantification showed that sLN moLCs expressed similar levels of CCR7 to eLCs, and to migratory, but not resident DC1s (Fig. 3*E*). To evaluate the role of CCR7 in moLCs, we generated a *Ccr7^f/f^* mouse that was crossed to *Cd11c*-Cre to generate a conditional knockout (Ccr7 cKO) (*SI Appendix*, Fig. S4 *A*–*D*). As expected, Ccr7 cKO mice harbor normal numbers of skin eLCs and dermal DC1s (*SI Appendix*, Fig. S4*E*). In sLNs, Ccr7 cKO mice completely lacked all migratory cells, including eLCs, migratory DC1s, and DC2s; however, lymphoid-resident DC1s and DC2s were still present, though decreased. To analyze the cell-intrinsic role of CCR7 in moLCs, we generated competitive BMC by transplanting 50% WT and 50% Ccr7 cKO bone marrow into lethally irradiated *Cd207*^DTR^ mice, followed by DT inoculation. WT and Ccr7 cKO bone marrow contributed equally to moLCs localized in the epidermis and dermis, as well as, lymphoid-resident DC1 and DC2 in sLN (Fig. 3 *F* and *G* and *SI Appendix*, Fig. S4*F*). However, only WT bone marrow contributed to migratory moLCs, and migratory DC1 and DC2 localized in sLN. These results indicate that CCR7 is necessary for moLC migration to sLN.

To confirm moLC migration from the skin to sLN, we assessed their capacity to carry a dye using the classic “TRITC skin painting” experiment. To control for cell-free dye diffusion, one ear was removed a few hours post-painting (ear excision). The peak of TRITC^+^ LCs in sLN was at 96 h, and it was blocked by ear excision (Fig. 3*H* and *SI Appendix*, Fig. S4*G*). moLCs carried a similar amount of TRITC to the auricular LN as eLCs, suggesting similar migration capabilities under the stimulatory conditions of the TRITC application. Contrarily to migratory LCs, lymphoid-resident DC1 were TRITC^-^ for the duration of the experiment (*SI Appendix*, Fig. S4*G*). We concluded that similar to eLCs, moLCs found in sLN are migrating from the skin.

### moLCs Are Intrinsically Superior at Migrating to sLN than eLCs.

**Fig. 4.**
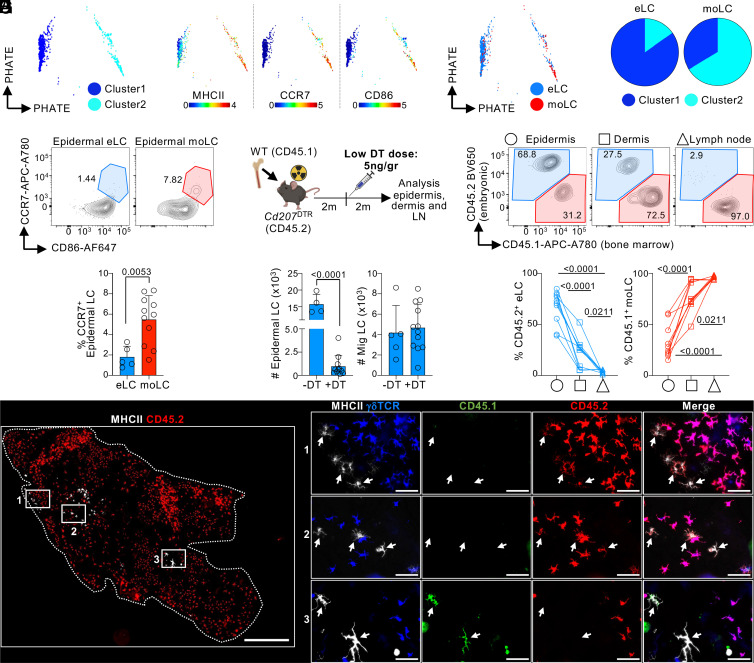
moLCs have a superior migratory capacity than eLCs. (*A*–*D*) B6 *Cd207*^DTR^ mice were inoculated or not with 50 ng/gr body weight DT, and whole skin was analyzed by CyTOF after 2 mo (1 of 2 exp.) (*A*) Skin LCs were gated as in S1A, analyzed by X-shift, and represented using PHATE. (*B*) PHATE map colored by the expression of the indicated marker (scale: ArcSinh). (*C*) Overlay of eLCs (−DT) and moLCs (+DT) onto the PHATE map of (*A*). (*D*) Frequency of eLCs (−DT) and moLCs (+DT) falling within cluster 1 and cluster 2 of the PHATE map (n = 2-4 in 2 exp.). (*E*) As in (*A*), but the epidermis was analyzed by flow cytometry for the expression of CCR7 and CD86 expression on eLCs (−DT) and moLCs (+DT). *Bottom*, frequency of CCR7^+^ LCs shown as the mean + SD of n = 5-11 mice (3 exp.; Student’s *t* test). (*F*−*H*) B6 CD45.2 *Cd207*^DTR^ mice were lethally irradiated and transplanted with CD45.1 WT bone marrow, followed by inoculation of a low dose of 5 ng/gr body weight of DT. Tissues were collected 2 mo later. (*F*) *Top*, experimental design. *Bottom*, number of epidermal LCs (*Left*) and Mig LCs in sLN (*Right*) as mean + SD (n = 4-12 in 3 exp.; Student’s *t* test). (*G*) *Top*, expression of CD45.1 and CD45.2 by flow cytometry. *Bottom*, frequency of CD45.2 eLCs and CD45.1 moLCs in each tissue shown as individual mice (n = 12 mice in 3 exp.; one-way ANOVA with Tukey’s multiple comparison). (*H*) Microscopy analysis showing CD45.1 and CD45.2 expression in epidermal LCs (1 of 2 exp.). *Left* panel, epidermal sheet stitching (scale bar: 500 μm); *Right* panels, magnification of white squares (scale bar: 50 μm).

We then asked whether moLCs have a different capacity to migrate to sLN than eLCs. We compared the phenotype of moLCs vs. eLCs localized in the skin by CyTOF analysis of *Cd207*^DTR^ ± DT (Fig. 4*A*). Gated skin LCs were clustered using X-shift and data represented by PHATE, a visualization tool that preserves local and global distances (59). Two clusters of skin LCs expressing CD326, CD24, and CD172α were distinguished (Fig. 4*A* and *SI Appendix*, Fig. S5*A*). Cluster 2 had higher expression of markers related to migration and activation, including CCR7, MHCII, and CD86 (Fig. 4*B*). Most moLCs (+DT) were within Cluster 2, whereas most eLCs (−DT) fell within Cluster 1 (Fig. 4 *C* and *D*). Analysis of only the epidermis showed similar results, i.e., higher expression of CCR7 and other activation markers by moLCs vs. eLCs (Fig. 4*E* and *SI Appendix*, Fig. S5*B*). Thus, a higher frequency of moLCs express migratory/maturation markers.

Until this point, our analysis of moLC was dependent on the complete elimination of eLCs by DT inoculation in *Cd207*^DTR^ mice. To control for off-target effects of systemic DT administration and to evaluate the intrinsic migratory capacity of moLCs, we designed an experimental model in which both eLCs and moLCs were present simultaneously in the epidermis. Thus, we inoculated a low dose of DT into *Cd207*^DTR^ BMC mice. We reasoned that this approach would allow the depletion of some, but not all eLCs, and the consequent repopulation with some moLCs. Low-dose DT inoculation resulted in epidermal LC depletion similar to our previous observations, and the complete repopulation of LCs in the sLN by 2 mo (Fig. 4*F*). Analysis of epidermal LCs showed ~60% were embryo-derived (CD45.2^+^) and ~40% were bone marrow–derived (CD45.1^+^) (Fig. 4*G*). Microscopy analysis confirmed the flow data by revealing patches of CD45.2^+^ eLCs and patches of bone marrow–derived CD45.1^+^ LCs (Fig. 4*H*), showing that eLCs and moLCs can coexist in the epidermal niche if a low dose of DT is used. Parallel flow cytometry analysis of the epidermis, dermis, and sLN was performed to compare the intrinsic migratory behavior of eLC vs. bone marrow–derived moLC (Fig. 4*G*). Dermis showed a greater frequency of LCs derived from bone marrow, although embryonic LCs were also present. In sLN, almost all LCs were derived from bone marrow. We concluded that in a competitive setting, bone marrow–derived moLCs have an intrinsic superior capacity to migrate to sLN than eLC.

### CD207-Dependent Capture of *Staphylococcus aureus* by moLCs Modulates Their Superior Migratory Capabilities.

**Fig. 5.**
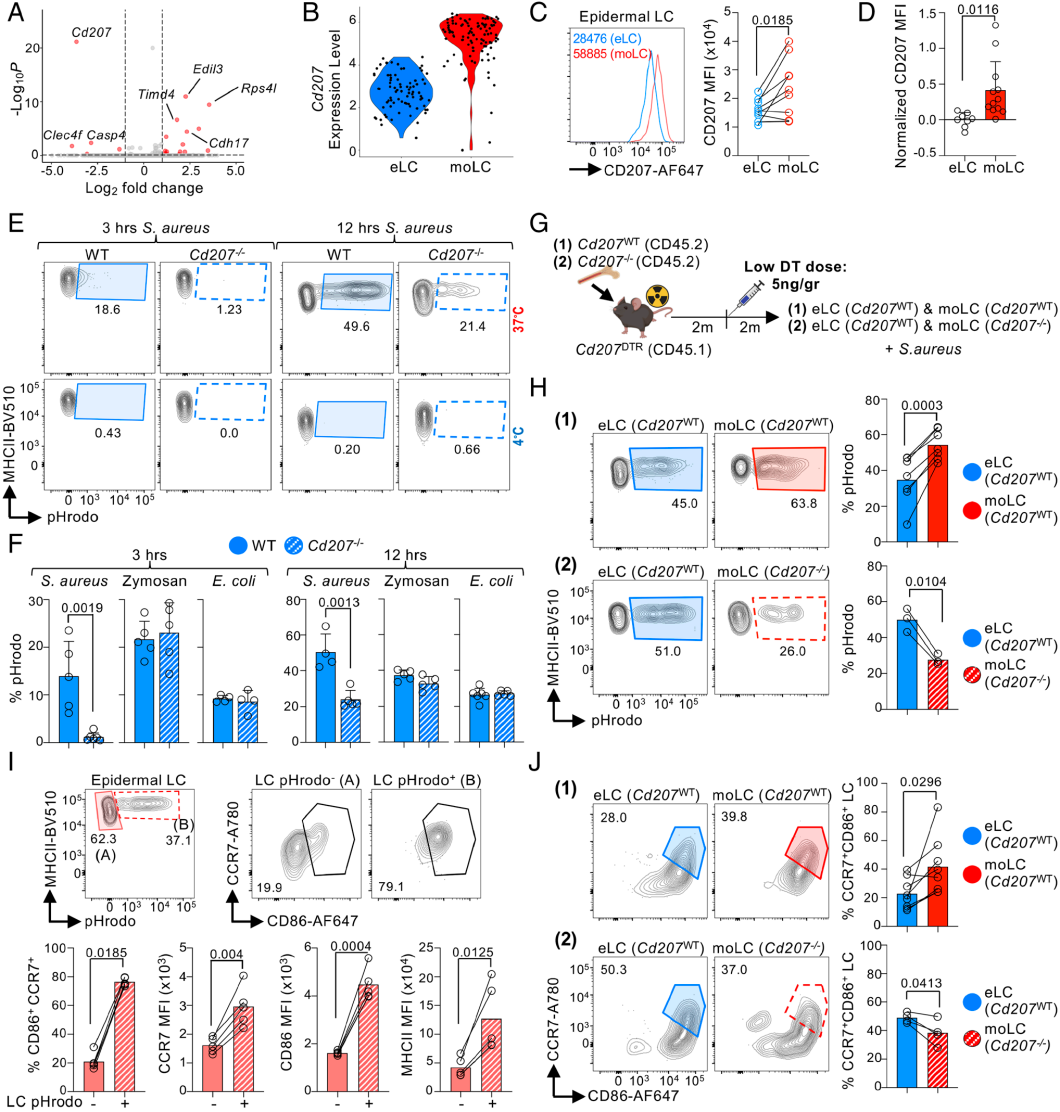
moLCs have a higher capacity to capture *S. aureus* through CD207. (*A*–*C*) CD45.1 *Cd207^DTR^* mice were lethally irradiated and transplanted with CD45.2 WT bone marrow. Two months later, a low dose of 5 ng/gr body weight DT was inoculated. (*A*) Epidermal eLCs and moLCs were purified and analyzed by SMART-seq2 2 mo post-DT. Volcano plot showing differentially expressed genes between eLCs and moLCs (log2 fold change = 1; *P*-value < 0.05). (*B*) As in (*A*), but Violin plot showing *Cd207* gene expression. (*C*) CD207 protein expression by flow cytometry. *Left*, representative histogram. *Right*, MFI expression (n = 9 mice in 3 exp.; Student’s paired *t* test). (*D*) *Cd207^DTR^* mice were inoculated or not with 50 ng/gr of body weight DT, and the epidermis was analyzed 2 mo later. CD207 expression on eLCs (−DT) and moLCs (+DT) shown as normalized MFI + SD (n = 8-12 mice in 3 exp.; Student’s *t* test). (*E* and *F*) Epidermis cell suspensions of WT B6 and *Cd207*^−/−^ mice were incubated with pHrodo-labeled microbes. (*E*) Flow cytometry plots showing the uptake of *S. aureus* by epidermal LCs after 3 (*Left*) and 12 (*Right*) hours of culture. (*F*) Bar plots showing uptake as the mean + SD (n = 4-6 in 2 to 3 exp.; Student’s *t* test). (*G* and *H*) CD45.1 *Cd207^DTR^* mice were lethally irradiated and reconstituted with CD45.2 WT (1) or CD45.2 *Cd207^−/^*^−^ (2) bone marrow. Two months later, a low dose of 5 ng/gr body weight DT was inoculated. Epidermis cell suspension was incubated with pHrodo *S. aureus* for 9 to 12 h. (*G*) Schematic of the experimental design. (*H*) *S. aureus* uptake in experimental setups (1) and (2), respectively. Representative flow cytometry plots (*Left*) and the bar graph of the mean + SD (n = 3-7 mice in 2 to 4 exp.; Student’s paired *t* test). (*I*) WT epidermis cell suspension was incubated with *S. aureus* for 9 to 12 h and then stained for migration markers. *Top*, flow cytometry plots in pHrodo^−^ and pHrodo^+^ LCs. *Bottom*, %CCR7^+^CD86^+^ and MFI of CCR7, CD86, and MHCII, shown as the mean + SD (n = 5 mice in 3 exp.; Student’s paired *t* test). (*J*) As in (*G*) but epidermal cell suspensions were analyzed by flow cytometry for CCR7 and CD86 expression after 6 to 9 h incubation with *S. aureus* (n = 4-7 in 3 to 4 exp.; Student’s paired *t* test).

To identify intrinsic differences between epidermal moLCs vs. eLCs, we performed transcriptomic analyses. Epidermal moLCs and eLCs were single cell sorted from BMC of *Cd207*^DTR^ mice inoculated with a low dose of DT and analyzed by SMART-seq2 (*SI Appendix*, Fig. S6 *A* and *B*). Unbiased analysis was unable to subcluster moLCs vs. eLCs (*SI Appendix*, Fig. S6*C*), suggesting a minor influence of origin to gene expression. Indeed, only very few genes were differentially expressed between eLCs and moLCs (Fig. 5*A*). Among these, eLCs expressed higher levels of the embryo-derived gene *Timd4* (17, 60, 61) (Fig. 5*A* and *SI Appendix*, Fig. S6*D*). There were no transcriptomic differences in migration and maturation genes between moLCs vs. eLCs, possibly because of low sequencing depth. However, epidermal moLCs expressed significantly higher levels of *Cd207* (Fig. 5*B*), which was confirmed at the protein level (Fig. 5*C*). Higher CD207 expression in moLCs was also observed when *Cd207*^DTR^ mice were inoculated with a high dose of DT (Fig. 5*D*), an experimental setting in which repopulating moLCs express the DTR transgene at similar levels to eLCs. Notably, moLC and eLC have different levels of CD207 only when localized in the epidermis and not sLNs (*SI Appendix*, Fig. S6*E*).

Human CD207 was recently described as a *S. aureus* uptake receptor (62). Mouse CD207 shares >70% identity with human CD207; however, it was reported to have weaker binding to *S. aureus* than its human counterpart (62). Nevertheless, these binding experiments were performed in vitro using soluble receptors, and the capacity of mouse CD207^+^ LCs to mediate *S. aureus* uptake has not been assessed yet. We incubated epidermal cell suspensions of WT and *Cd207*^−/−^ mice with *S. aureus* conjugated with pHrodo, a pH-sensitive fluorogenic dye that allows the discrimination of endocytosed from adherent particles. At both 3 and 12 h, WT epidermal LCs showed a greater capacity to capture *S. aureus* than *Cd207*^−/−^ epidermal LCs, at 37 °C but not 4 °C (Fig. 5*E*). WT and *Cd207*^−/−^ LCs captured pHrodo-conjugated Zymosan and *Escherichia coli* with comparable efficacy (Fig. 5*F*). Similar to human CD207, mouse CD207 has affinity for *S. aureus*.

Given that epidermal moLCs expressed higher levels of CD207, we asked whether these cells have a differential capacity to capture *S. aureus*. BMC of *Cd207*^DTR^ mice were inoculated with a low dose of DT to permit for the simultaneous presence of epidermal eLCs and moLCs, and the uptake of *S. aureus* was evaluated in epidermal cell suspensions by flow cytometry using pHrodo- conjugated bacteria (Fig. 5*G*). In the first set of experiments, WT bone marrow was transplanted into *Cd207*^DTR^ mice to allow for repopulation with CD207-expressing moLCs. We observed that a higher frequency of moLCs vs. eLCs captured pHrodo-conjugated *S. aureus*, in accordance with their higher CD207 expression (Fig. 5*H*). In a second set of experiments, *Cd207*^−/−^ bone marrow was transplanted into *Cd207*^DTR^ mice to allow for repopulation with CD207-deficient moLCs. CD207-deficient moLCs were inferior to CD207-expressing eLCs at capturing pHrodo-conjugated *S. aureus* (Fig. 5*H*). Thus, epidermal moLCs differ from eLCs in the expression of CD207 and ability to uptake *S. aureus*.

We next asked whether microbe uptake through CD207 could promote the upregulation of the migration receptor CCR7. Analysis of a WT epidermal cell suspension incubated with *S. aureus* showed that pHrodo^+^ LCs had higher expression of CCR7, and activation markers (CD86, MHCII), than pHrodo^-^ cells (Fig. 5*I*). Using the experimental setup as in Fig. 5*G*, we then compared the upregulation of migration markers by eLCs and moLCs. A higher frequency of moLCs expressed CCR7 and CD86 than eLCs after challenge with *S. aureus* (Fig. 5*J*). The upregulation of migration/maturation markers by moLCs was decreased when these cells lacked CD207. We concluded that higher expression of CD207 by moLCs permits a greater uptake of skin microbes, which in turn increases CCR7 and migration to sLN. Notably, we did not observe an overall defect in the capacity of *Cd207*^−/−^ moLCs to migrate to sLN in vivo (*SI Appendix*, Fig. S6*F*), suggesting that other uptake receptors also play a role in modulating moLC migration.

## Discussion

It is still debated whether bone marrow monocyte-derived tissue macrophages can acquire all the transcriptional and functional features of embryo-derived macrophages. Current evidence suggests that ontogeny accounts for heterogeneity in the features of some tissue macrophages, but not others (17, 18, 20, 63, 64). Here, we show that monocytes seeding the epidermis are able to acquire most of the features of eLCs. However, moLCs are more migratory than eLCs. Mechanistically, we show that moLCs express higher levels of CD207 for the uptake of *S. aureus*, which in turn promotes the upregulation of the chemokine receptor CCR7 for migration to sLN.

The contribution of distinct progenitors to tissue macrophages has been previously analyzed and is tissue specific (11, 12). This tissue-specific heterogeneity is regulated by different mechanisms including niche accessibility, quorum sensing, and inflammatory cues (3). Whereas embryonic progenitors can access the epidermis during embryogenesis (22, 23), bone marrow monocytes may only have partial accessibility in the absence of inflammation. This is in contrast to the liver, where monocytes quickly infiltrate and differentiate into Kupffer cells in the absence of inflammation (17). eLCs and moLCs may also have different quorum-sensing mechanisms. eLC density in the epidermis may be controlled by CSF-1 or IL-34 (44), and eLC maintenance is regulated by autocrine/paracrine TGFβ (51, 65–67). In contrast, our data suggest that moLCs may be sensing cues from the sLN. Specifically indicative is our observation that Ki67^+^ epidermal moLCs peak at 2 mo, a time point in which the sLN niche is being filled. After the sLN niche is filled (>2 mo), moLC proliferation goes back to normal levels even though the epidermal niche is still empty. More studies are needed to understand the signals that LCs are using to balance their numbers in the skin and sLN at steady state.

Inflammatory conditions are also key in determining the contribution of monocytes to LC heterogeneity. Different from steady-state conditions, moLCs are able to repopulate the epidermis in various inflammatory models (27, 42, 68, 69), as observed here using tape striping. Recent work from Bennett’s group has shown that during GVHD, monocytes infiltrate the epidermis, but inefficiently convert into moLCs, resulting in the accumulation of an intermediate partially differentiated cell state (44). Thus, the presence or not, as well as the type of inflammation are key for epidermal LC repopulation. Importantly, moLCs can acquire all the transcriptomic features of eLCs during GVHD (44). It is still unclear why at steady state moLCs are unable to acquire every transcriptomic feature of eLCs, but duration of residency in the tissue may be an important factor (2). Indeed, *Timd4*, a gene associated with long-term residence in the gut and liver (17, 60), is expressed at higher levels in eLCs vs. moLCs. Further work is needed to dissect whether ontogeny modulates other features of LCs besides their migration to sLN.

*S. aureus* is associated with *s*kin infections (70, 71), and the receptor for this skin bacterium was recently described to be human CD207; however, it was unclear whether this mechanism of bacterial uptake is conserved between species. Here, we demonstrate that mouse CD207 also has affinity for *S. aureus*. It has been suggested that *S. aureus* is transferred from humans and becomes a mouse commensal in animal facilities (72), although this was not formally tested in our study. Alternatively, CD207 may have affinity for other Staphylococcal genera present in mice. Either way, we show that the differential expression of CD207 by moLCs vs. eLCs regulates the capacity of these cells to uptake *S. aureus* and up-regulate CCR7. Notably, CD207-mediated capture of *S. aureus* is probably one of various mechanisms that promotes migration, as we did not observe a defect in the number of cells in sLN when analyzing *Cd207*^−/−^ moLCs. Nevertheless, our data support the conclusion that distinct intrinsic pathways modulate moLC migration to sLN.

The capacity of epidermal LCs to migrate through the afferent lymphatics of the skin to draining LN was originally noted by Siberberg-Sinakin et al. in response to contact allergens (73). A vast amount of literature supports the original observations that LCs are able to exit the epidermis and migrate to sLN (28, 74). Recently, Sheng et al. challenged this paradigm and suggested that a dermal-resident LC-like, but not epidermal eLCs, is the migratory population that traffics to sLN (36). The study by Sheng et al. relied heavily on the expression of a few markers to delineate LC subpopulations, e.g., CD11b and F4/80; however, these markers may not be a reliable readout since LC phenotype changes as they migrate to sLN (75). Thus, it is not clear whether LC-like described by Sheng et al. are merely eLCs migrating through the dermis. Alternatively, LC-like may be moLCs. Indeed, if eLCs are occupying the epidermal niche (e.g., in the absence of partial/total depletion), monocytes would inefficiently enter the epidermis and only few cells would differentiate into moLCs; instead, moLCs would be found in the dermis and sLN given their superior migratory capability. Accordingly, Sheng et al. reported that ~50% of migratory eLCs are replaced by bone marrow–derived LCs in the sLN, but not the epidermis, of BMC mice. In our BMC, we do not observe a loss of migratory eLCs at any time point analyzed unless these cells are depleted by DT. This discrepancy may be dependent on extrinsic vivarium-specific conditions that are permissive to moLC infiltration into the skin following irradiation. Notably, Sheng et al. show that eLCs express different C-type lectin receptors than LC-like, similar to our observations between eLCs vs. moLCs. Future studies will aim at systematically assessing C-type lectin expression, and harnessing these receptors for the delivery of vaccine compounds to eLCs vs. moLCs (29).

The superior migratory feature of moLCs may play a major role in the maintenance of an intact sLN niche when LC numbers dwindle, e.g., during aging (76, 77). Accordingly, LCs of aged mice express higher levels of CD207 (76), suggesting a monocytic origin. Monocyte tracing models, like *Ms4a3*^Cre^ x*Rosa26*^LSL-TdTomato^, may offer insights into the functional diversification between eLCs and moLCs during immunosenescence. These studies have the potential to unravel therapeutic strategies that enhance skin immune function during aging.

In summary, our study sheds light on the dynamic repopulation of skin LCs in the steady state and reveals the distinct migratory capacity of moLCs.

## Materials and Methods

### Mice.

Wild-type (WT) C57BL/6 CD45.2 (B6; Jax#000664) and CD45.1 (NCI B6-Ly5.1 Cr#564) mice were purchased from Jackson Laboratory and Charles River, respectively. *Cd207^DTR^* and *Cd207^EGFP^* (50) mice were obtained from Malissen and bred in-house as homozygous CD45.2 or CD45.1. *Cd207^DTR^* were crossed for >10 generations to Balb/c*. Cd207^−/−^* mice were generated by breeding *Cd207^Cre^* mice as homozygotes (39). *Ms4a3^Cre^* [C57BL/6J-*Ms4a3^em2(cre)Fgnx/J^*; Jax#036382] were bred to *Rosa26*^LSL-TdTomato^ [B6.Cg-*Gt(ROSA)26Sor^tm9(CAG-tdTomato)/Hze^*; Jax#007909] in-house. *hCd207*-Cre mice were generously provided by Kaplan (51) and bred to *Rosa26*^iDTR^ [C57BL/6-*Gt(ROSA)26Sor^tm1(HBEGF)Awai^*; Jax#007900]. *LysM*-Cre [B6.129P2-*Lyz2tm1(cre)Ifo;* Jax#004781] were bred to *Csf1r*^LSL-DTR^ [C57BL/6-Tg(Csf1r-HBEGF/mCherry)1Mnz; Jax#024046]. *Ccr7^f/f^* mice were generated at the Stanford Medicine Transgenic, Knockout, and Tumor Model Center (TKTC). Murine B6 zygotes were injected with single guide RNA (sgRNA) (*SI Appendix*, Fig. S4*C*), Cas9 protein (*Streptococcus pyogenes* Cas9, Integrated DNA Technologies), and a donor DNA construct containing homologous arms and loxP sites around Ccr7 exon 3 (Biomatik, *SI Appendix*, Fig. S4*A*). Pups were screened by PCR (*SI Appendix*, Fig. S4 *C* and *D*). Transgenic mice were backcrossed for two generations to WT B6 mice. Mice were then crossed to *Cd11c*-Cre mice [B6.Cg-Tg(*Itgax-cre*)1-1Reiz, Jax#08068] and screened for *Ccr7^wt/wt^*, *Ccr7^f/wt^*, *Ccr7^f/f^*, and the appearance of germline deletion by PCR (*SI Appendix*, Fig. S4 *C* and *D*). Diphtheria toxin (50 ng/gr body weight; Sigma) was injected retro-orbitally, unless otherwise noted. For BMC, mice were lethally irradiated with 12 Gy (2 doses of 6 Gy administered 3 h apart) and transplanted with 3 × 10^6^ bone marrow (BM) cells. In some experiments, the head and shoulders of mice were shielded from a 9 Gy radiation dose using a lead cover. Water was supplemented with 40 mg/mL sulfamethoxazole and 8 mg/mL trimethoprim (Aurobindo Pharma) for 2 wk following bone marrow transplantation. For tape-stripping, mice were anesthetized using isoflurane, and the ventral and dorsal sides of both ears were stripped 12 times with Scotch™ tape (3M) (78).

### Cell Suspensions.

For whole skin cell suspension, ears were removed, separated into ventral and dorsal halves, and incubated with a solution of 1.3U/mL Liberase TL (Roche) and 50 µg/mL DNase I (Roche) in plain RPMI (Corning) for 45 min at 37 °C. Tissue was then minced and incubated for another 45 min with continuous shaking. Enzymatic digestion was stopped by the addition of 5 mM EDTA (Corning) for 5 min. For epidermis and dermis cell suspensions, ears were separated into ventral and dorsal halves and incubated with 0.4 mg/mL of Dispase (Roche) in Hank’s balanced salt solution (HBSS) buffer with Ca^2+^ and Mg^2+^ (Corning) for 150 min. The epidermis was separated from the dermis using thin forceps, and both tissues were minced and incubated with 1.5 mg/mL Collagenase IV (Worthington Biochemical) and 70 µg/mL DNase I in R2 [RPMI with 2% fetal bovine serum (FBS) (Gibco), 1× penicillin/streptomycin (Corning), and 2 mM HEPES buffer (Corning)] for 90 min at 37 °C. For epidermal back skin cell suspensions, hair was removed by shaving and application of Nair™ Hair Remover lotion (Church and Dwight Co.) for 5 min., followed by fat removal using scalpels. Back skin was incubated for 2 h in 2.5 mg/mL Dispase in HBSS buffer with Ca^2+^ and Mg^2+^ at 37 °C. The epidermis was removed with thin forceps, minced, and incubated for 2 h with 400 U/mL Collagenase D (Sigma) and 50 µg/mL DNase I in R10 [(RPMI with 10% FBS, 1× penicillin/streptomycin, and 1× glutamine (Corning)] at 37 °C. In all cases, enzymatic digestion was stopped with 10 mM EDTA, and cells were filtered with a 100 µM cell strainer. For skin-draining lymph nodes cell suspensions, popliteal, inguinal, axillary, and brachial lymph nodes (sLN) were harvested, minced, and incubated with 400 U/mL Collagenase D and 5 µg/mL DNase I in HBSS buffer with Ca^2+^ and Mg^2+^ for 30 min at 37 °C. Then, 10 mM EDTA was added in the final 5 min of the digestion. In some cases, one auricular LN was harvested for analysis, minced, and incubated with 2.5 µg/mL Liberase TL and 50 µg/mL DNase I for 30 min. Then, 10 mM EDTA was added in the last five min of digestion. LN cells were filtered through a 70 µm strainer before analysis.

### Flow Cytometry.

Cell suspensions were incubated in anti-(α-) CD32/CD16 (clone 2.4G2; prepared in-house) for 10 min at 4 °C followed by staining antibodies (Abs) against surface markers (*SI Appendix*, Table S1) in flow cytometry buffer (PBS plus 2% FBS and 2 mM EDTA) at 4 °C for 20 min. Unless otherwise specified, lineage markers were CD3, CD19, and NK1.1. Cells were fixed using either BD cytoplasmic fixation kit (BD Biosciences) for cytoplasmic proteins or with the Foxp3 Transcription Factor Fixation kit (Thermo Fisher Scientific) for Ki67 staining according to the manufacturer’s instructions. Abs against intracellular markers were added for 30 to 45 min. Fluorescence minus one (FMO) and isotype controls were used to control for specific staining. Samples were acquired on a LSRFortessa X-20 (BD Bioscience) and analyzed using FlowJo software (FlowJo).

### CyTOF.

Skin and sLN cell suspensions were stained for CyTOF analysis as previously described (79). Briefly, cell suspensions were stained for 5 min at RT with 0.25 µM cisplatin (Fluidigm). Samples were washed with CyFACS buffer [1% bovine serum albumin (BSA) and 2 mM EDTA in PBS] and barcoded with CD45 antibodies, pooled, and stained with the CyTOF surface Ab cocktail for 30 min at 4 °C (*SI Appendix*, Table S2). Cells were washed, fixed for 20 min with a BD cytoplasmic fixation kit, and stained for intracellular Abs for 30 min at 4 °C. Samples were fixed with 2% fresh paraformaldehyde (Electron Microscopy Sciences) and 125 nM Iridium intercalator (Fluidigm) overnight. Samples were resuspended in water, filtered, and analyzed on a CyTOF2 (Fluidigm). For analysis, fcs files were normalized using ParkerICI/Premessa R. Lineage^+^ cells (CD3, CD19, CD335, and Ly6G) were excluded, and UMAP, PHATE, and X-shift analysis was performed on the remaining myeloid cells using FlowJo (FlowJo). Data visualization was done using CYT (SightOf) in MATLAB (MathWorks) (80). Heatmaps of protein expression were generated using the Mean Signal Intensity (MSI).

### Microscopy.

Hair of the ears was removed using Nair™ Hair Remover lotion for 2 min and incubated in 0.5 M EDTA at 37 °C for 2 h, followed by 30 min in PBS. Epidermal sheets were then separated from the dermis using thin forceps followed by fixation with cold acetone for 20 min. Epidermal sheets were blocked with an Avidin/Biotin blocking kit (Life Technologies) followed by 0.2% BSA in PBS and stained with Abs (*SI Appendix*, Table S3). Isotype controls and secondary-only Abs were used to control for specific staining. Images were captured with a BZ-X800 fluorescence microscope (Keyence), and stitching was performed with microscope software (BZ-H4XD, Keyence). Imaging analysis was performed using ImageJ (version 1.54; NIH).

### TRITC Painting.

First, 50 mg/mL tetramethylrhodamine-5-(and-6)-isothiocyanate (TRITC) (Thermo Fisher) was resuspended in DMSO (Sigma) and diluted to 0.5% v/v in a solution of 1:1 acetone (Sigma)/dibutylphthalate (Sigma). Then, 10 µL of this final solution was applied to the dorsal side of one ear and allowed to dry for 10 min. After 96 h, mice were euthanized according to procedures approved by the Stanford University Administrative Panel on Laboratory Animal Care (APLAC).

### Phagocytosis Assays.

Epidermal cell suspensions were incubated with 10 µg/mL pHrodo red *S. aureus* bioparticles (Thermo Fisher), 100 µg/mL pHrodo red *Zymosan* bioparticles (Thermo Fisher), or 10 µg/mL *E. coli* bioparticles (Thermo Fisher) in R10 for 3 to 12 h at 37 °C or at 4 °C as an uptake control.

### scRNA-seq and Analysis.

B6 *Cd207^DTR^* CD45.1 mice were transplanted with WT CD45.2 bone marrow, followed by inoculation of 5 ng/gr body weight DT. Two months later, CD45^+^ cells were purified from the epidermis using α-CD45-biotin antibody (clone: 30-F11) and α-biotin beads (Miltenyi). Cells were single-cell sorted on a Fluorescence-Activated Cell Sorting (FACS) Aria Fusion (BD Biosciences). Lineage^−^(CD3^−^/CD19^−^/Nk1.1^−^/Ly6G^−^/SiglecF^−^)/CD11c^+^/CD11b^+^/CD24^+^/MHCII^+^/CD326^+^ LCs were index sorted using congenic markers (CD45.2 and CD45.1). The Takara Smart-Seq Single Cell kit was used for reverse transcription, cDNA synthesis, and amplification (Takara). Ampure XP beads (Beckman Coulter) were used for cDNA cleanup. Quantification was performed using the Quant-iT PicoGreen dsDNA Assay kit (Thermo Fisher). The Illumina Nextera XT DNA Library Prep and Unique Dual Index Kits (Illumina) were used for library prep. Paired-end 75 bp sequencing was performed on a HiSeq 4000 (Illumina). Alignment was performed using STAR to the mouse reference genome mm10. A total of 185 cells were sequenced and analyzed using Seurat (v.5.0.1 Satija Lab). Cells with more than 5% mitochondrial genes were filtered out, resulting in 182 high-quality cells. Data were normalized and scaled using “LogNormalize” method (scale factor 10,000) and “ScaleData” function from Seurat. Genes differentially expressed between CD45.1 and CD45.2 LCs were identified using the Wilcoxon rank-sum test implemented in Seurat’s FindMarkers function.

### Statistical Analysis.

Statistical analysis was performed by Prism software (GraphPad) using unpaired Student’s *t* tests for groups of two (unless otherwise specified) and one-way ANOVA with Tukey’s post hoc test or Dunnett’s multiple comparison for others. *P*-values are considered significant when <0.05. Heatmaps were generated using the Broad Institute’s software Morpheus, https://software.broadinstitute.org/morpheus.

## Supplementary Material

Appendix 01 (PDF)

## Data Availability

The single cell RNA sequencing data reported in this paper have been deposited in the Gene Expression Omnibus (GEO) database, www.ncbi.nlm.nih.gov/geo (accession no. GSE261079) (81). The mass cytometry data has been deposited at the FlowRepository database, http://flowrepository.org/ (accession no. FR-FCM-Z785) (82). This study did not generate a unique code. The *Ccr7*^f/f^ mice can be requested and will be provided by J.I.
