# Supplementary material for: Monocytes give rise to Langerhans cells that preferentially migrate to lymph nodes at steady state
Source: Proc Natl Acad Sci U S A. 2024 Nov 14;121(47):e2404927121. doi: 10.1073/pnas.2404927121 (PMC11588065; doi:10.1073/pnas.2404927121)
Supplement: Supplementary file 1 — Appendix 01 (PDF) [file pnas.2404927121.sapp.pdf]

Supplementary Figure 1

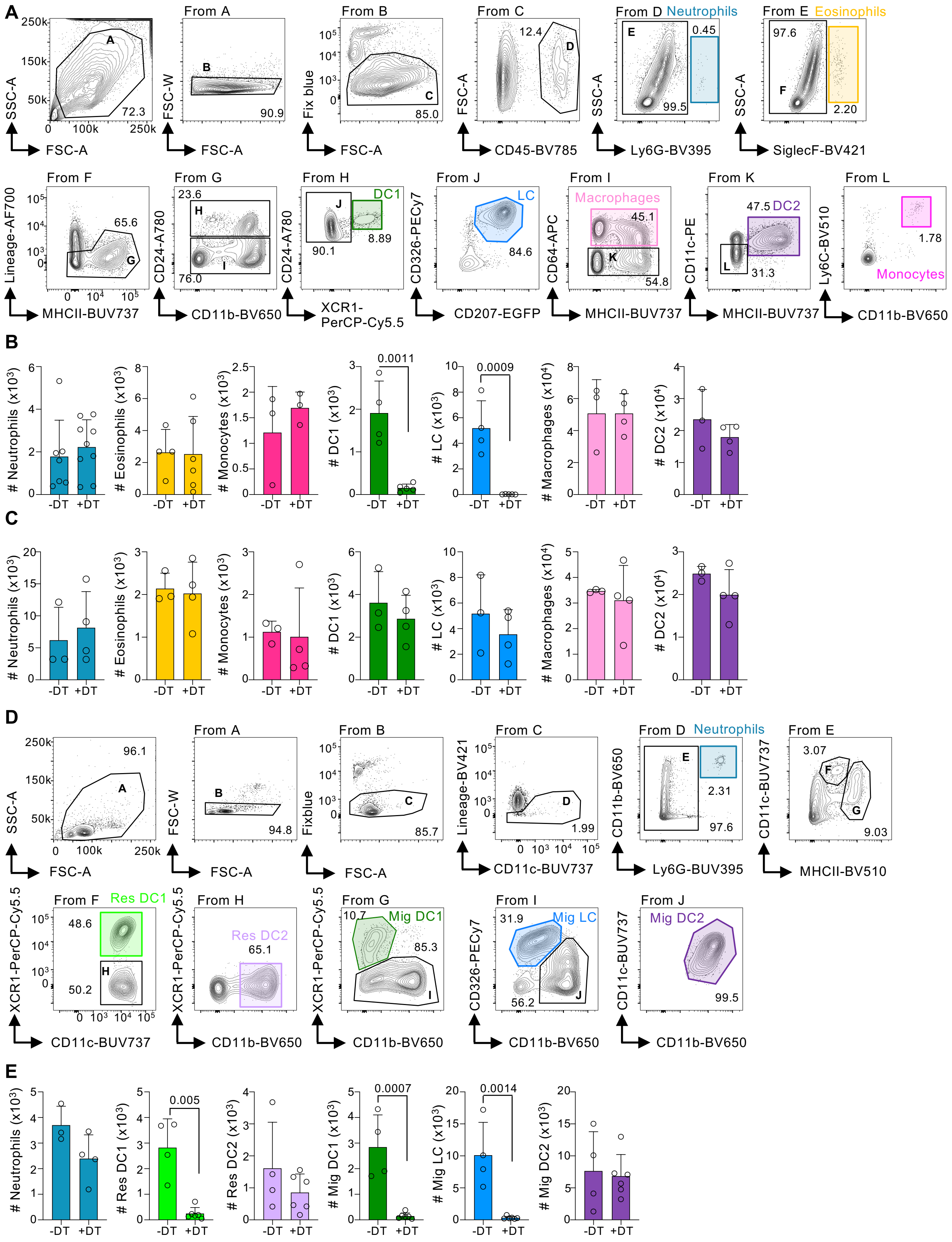

FIGURE S1. DT inoculation in *Cd207*$^{\mathbf{DTR}}$ mice specifically depletes LCs and DC1s, without causing inflammation. (A-B) Balb/c *Cd207*^{DTR} mice were injected with 50 ng/gr body weight DT (+DT) or left untreated (-DT), and 2 days later whole skin and sLN were analyzed by flow cytometry. **(A)** Gating strategy used for the identification of different myeloid cells in the skin. **(B)** Number of myeloid cells in the skin shown as the mean + SD (n= 3-9 mice in 2-4 exp.; Student's t-test). **(C)** B6 *Cd207*^{EGFP} mice were injected with 50 ng/gr body weight DT (+DT) or left untreated (-DT), and 2 days later whole skin was analyzed by flow cytometry. Number of myeloid cells in the skin shown as the mean + SD (n= 3-4 mice in 2 exp.; Student's t-test). **(D)** As in A, but sLN were analyzed. **(E)** As in B, but the number of myeloid cells in sLN shown as the mean + SD (n=3-6 mice in 3-4 exp.; Student's t-test).

Supplementary Figure 2

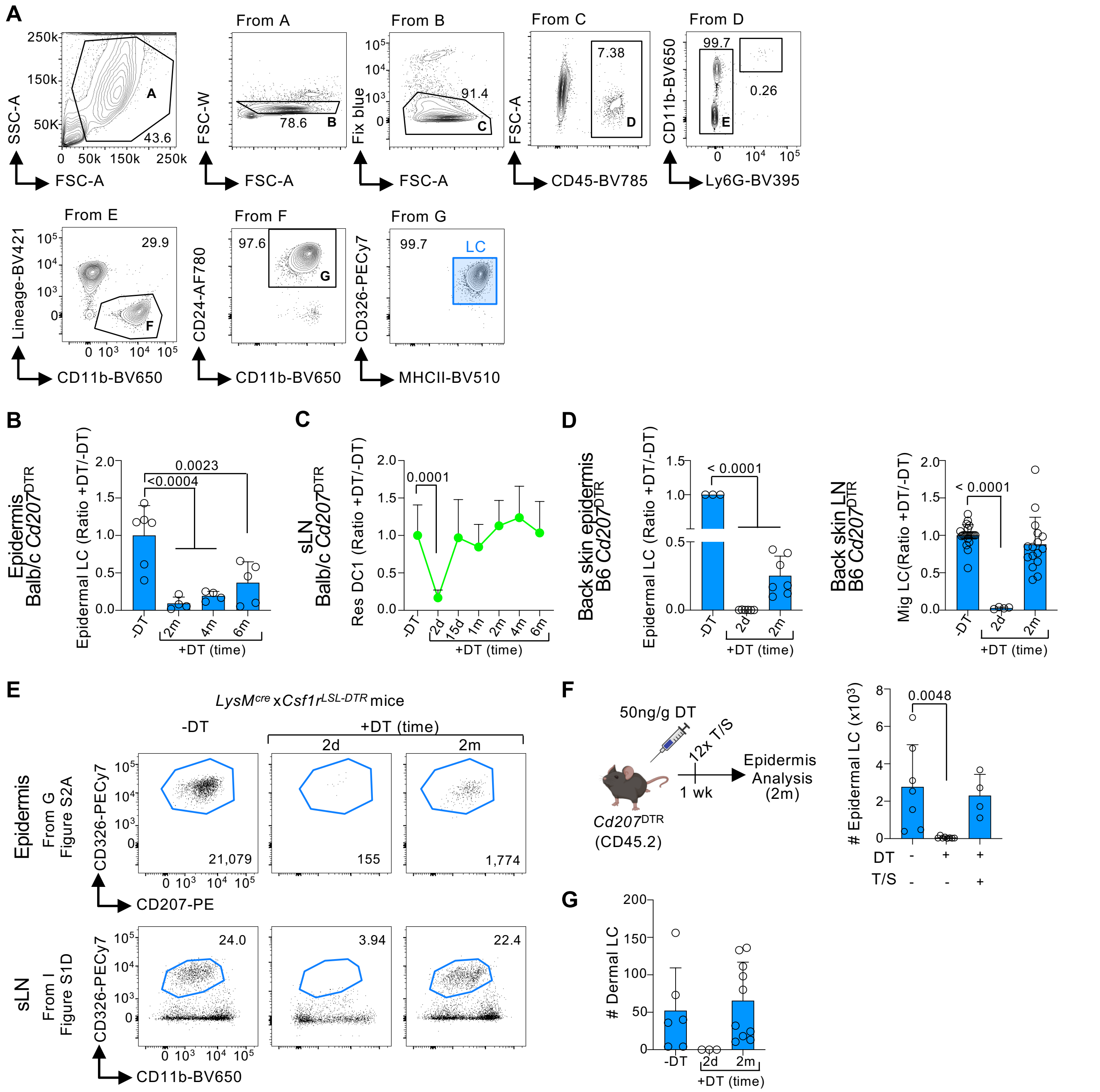

FIGURE S2. LCs repopulate sLN, but not epidermis, at steady state. (A-C) Balb/c *Cd207*^{DTR} were inoculated with 50 ng/gr body weight DT (+DT) or left untreated (-DT). Epidermis, dermis and sLN were collected and analyzed at different time points. **(A)** Gating strategy for the identification of LCs in epidermal cell suspensions (epidermal LCs). **(B)** Bar graph showing the mean + SD of epidermal LCs in Balb/c *Cd207*^{DTR} mice at different times post-DT inoculation, normalized to non-DT controls (n=4-6 mice in 2 exp.; One-way ANOVA with Dunnett's multiple comparison). **(C)** Mean + SD of resident DC1s in the sLN of Balb/c *Cd207*^{DTR} mice at different times post-DT inoculation, normalized to non-DT controls (n=5-18 mice in 4-10 exp.; One-way ANOVA with Dunnett's multiple comparison). **(D)** Left, bar graph showing the mean + SD of epidermal LCs obtained from the back skin of B6 *Cd207*^{DTR} mice, normalized to non-DT controls (n=3-7 mice in 3 exp.; One-way ANOVA with Dunnett's multiple comparison). Right, bar graph showing the mean + SD of Mig LCs in the back skin draining LN of B6 *Cd207*^{DTR} mice, normalized to non-DT controls (n=4-22 mice in 3-7 exp.; One-way ANOVA with Dunnett's multiple comparison). **(E)** *LysM*^{cre} x*Csf1r*$^{LSL-DTR}$ mice were injected with 50ng/gr body weight DT, and epidermis and sLN were collected 2 days (2d) or 2 months (2m) later. Top, representative flow cytometry plots of epidermal LCs. Bottom, representative flow cytometry plots of Mig LCs in sLN. **(F)** B6 *Cd207*^{DTR} were inoculated with 50ng/gr body weight DT. One-week post-DT inoculation, mice ears were tape-stripped 12 times (+T/S) or not (-T/S). Two months post-DT, epidermis was collected. Left, schematic of experimental design. Right, LC numbers shown as the mean + SD (n=4-8 mice in 2 exp.; One-way ANOVA with Dunnett's multiple comparison). **(G)** B6 *Cd207*^{DTR} were inoculated with 50 ng/gr body weight DT (+DT) or left untreated (-DT). Dermis was collected and analyzed at 2 days (2d) and 2 months (2m) post-DT. Bar graph showing the number + SD of dermal LCs (n=3-10 mice in 2-3 exp.; One-way ANOVA with Dunnett's multiple comparison).

Supplementary Figure 3

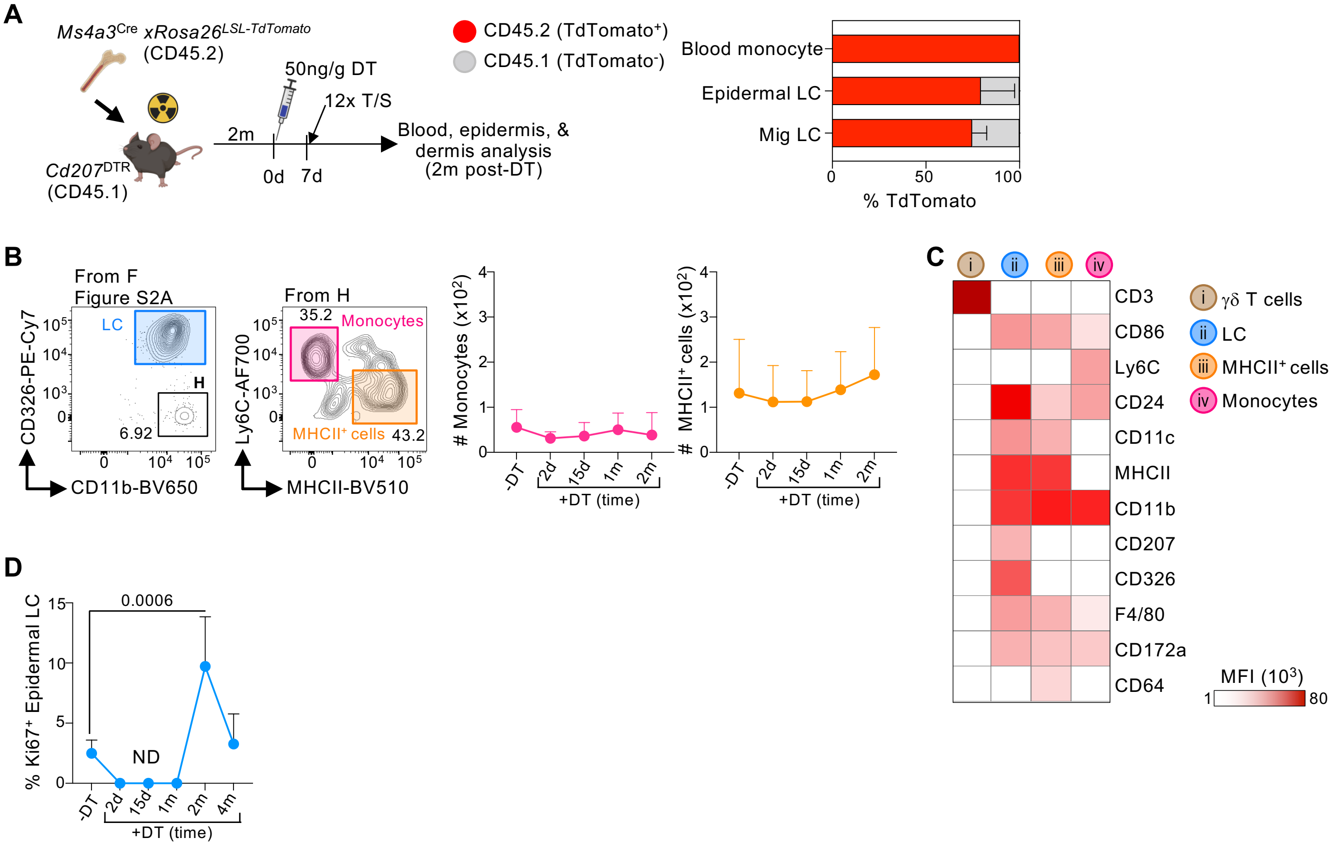

FIGURE S3. Few monocytes infiltrate the epidermis, and differentiate into moLCs that undergo proliferation. (A) B6 CD45.1 *Cd207*DTR mice were lethally irradiated and reconstituted with CD45.2 *Ms4a3*cre x *Rosa*LSL-TdTomato bone marrow. Two months later, 50ng/gr body weight DT was inoculated. One-week post-DT, mice were tape-stripped (T/S) 12 times, and epidermis, LN, and blood was collected 2 months after that. Left, experimental design. Right, bar graph showing mean + SD of TdTomato expression in each cell population, normalized to blood monocytes (n=3 mice in 1 exp.). **(B-D)** B6 *Cd207*DTR mice were treated (+DT) with 50ng/gr body weight DT, or not (-DT). **(B)** Left, gating strategy used to identify epidermal monocytes and MHCII+ cells. Right, number of epidermal monocytes and MHCII+ cells shown as the mean + SD (n=3-20 in 2-5 exp.; One-way ANOVA with Dunnett's multiple comparison). **(C)** Marker expression heatmap for each population of epidermal cells (n=2-3 mice in 1 exp.). **(D)** Frequency of Ki67+ epidermal LCs, shown as the mean +SD (n=3-9 mice in 2-3 exp.; One-way ANOVA with Dunnett's multiple comparison). ND: not determined; d: days; m: months.

Supplementary Figure 4

A

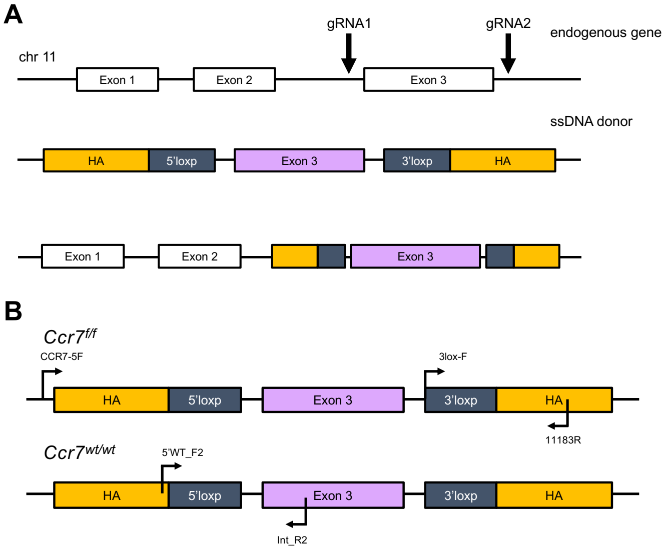

C

	Primer	Sequence (5'-3')	Product size
5' loxP Screening PCR	CCR7-5F	CACAGGAGTTGCGATTTGCCTC	897 bp
	CCR7-5lox-R	GGATCTCACAATAACTTCGTATAGCA TACA	
3' loxP Screening PCR	CCR7-3R	CCTGTGGGGATGGAGTGAGG	874 bp
	CCR7-3lox-F	TCCGGAGATGCAAACATAACTTCG	

gRNA	gRNA (5'-3')
1	ACAGTCCGGATCTCACAGTC
2	CTCTCCGGAGATGCAAACCC

	Primer	Sequence (5'-3')	Product size
3' loxP PCR	CCR7-3loxF	TCCGGAGATGCAAACATAACTTCG	flox (220)<br>KO (995)
	CCR7-5F	CACAGGAGTTGCGATTTGCCTC	
	CCR7-11183R	GTTCCCAGACACAGGAGTTCC	
WT PCR	CCR7-5'WT_F2	GTCCTGACTGTGAGAT	431
	CCR7-Int_R2	GCAGCATCCCGCTGAAGAAGC	

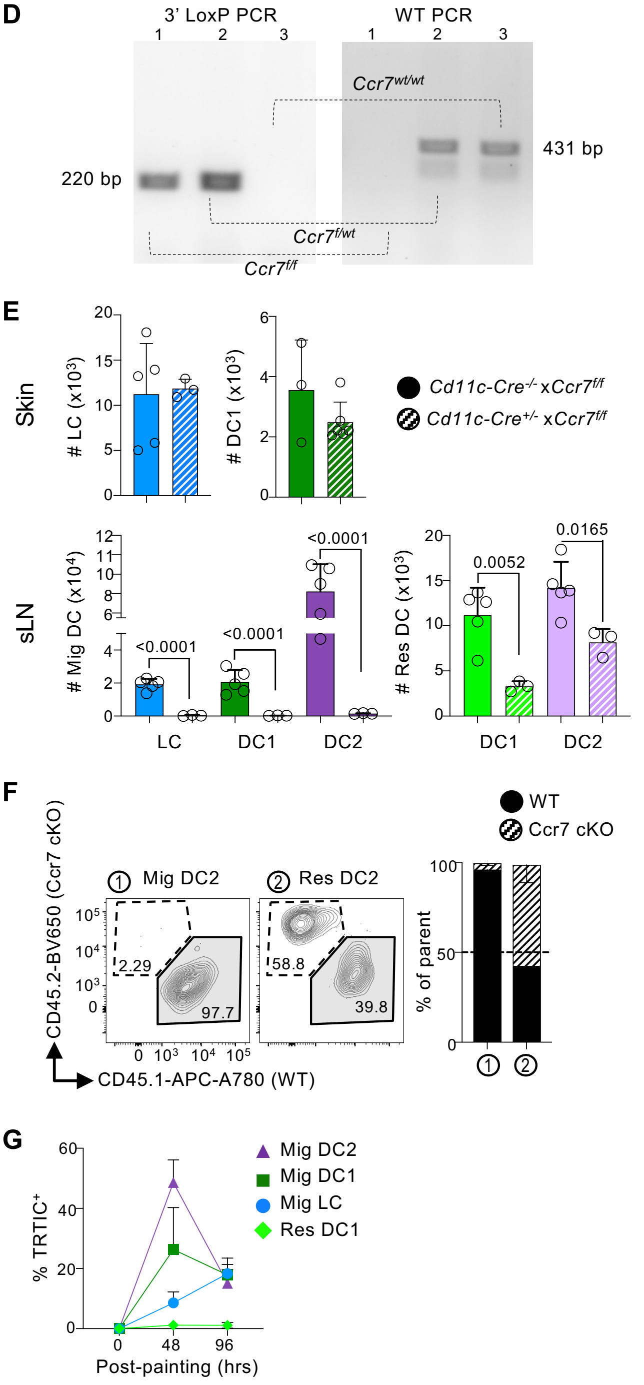

FIGURE S4. Generation of *Ccr7*$^{f/f}$ mouse. B6 murine zygotes were injected with sgRNA, Cas9 protein, and donor DNA containing homologous arms and loxp sites flanking exon 3 of the *Ccr7* gene. *Ccr7*$^{f/f}$ mice were bred to *Cd11c*-Cre mice to generate Ccr7 conditional knockout (Ccr7 cKO) mice. **(A)** Schematic showing CRISPR-Cas9 targeting strategy of exon 3 of the *Ccr7* gene (top), ssDNA donor DNA (middle), and final inserted transgene (bottom). **(B)** Schematic showing primer design for the detection of *Ccr7*$^{f/f}$ (top) and *Ccr7*$^{wt/wt}$ (bottom) mice. **(C)** Top, primer set used to screen founders for presence of inserted loxp sites. Middle, gRNA sequences used to target Cas9 for double stranded-breaks. Bottom, genotyping primers used to identify flox, WT, and germline deletions (KO). **(D)** Representative PCR showing examples of *Ccr7*$^{f/f}$ (1), *Ccr7*$^{f/wt}$ (2), and *Ccr7*$^{wt/wt}$ (3) genotyping results. **(E)** Skin and sLN of Ccr7 cKO (*Cd11c*-Cre$^{+/-}$ x*Ccr7*$^{f/f}$) and control (*Cd11c*-Cre$^{-/-}$ x*Ccr7*$^{f/f}$) mice were collected for myeloid cell quantification. Top, epidermal LCs (left) and dermal DC1s (right) shown as the mean + SD (n=3-6 mice in 3 exp.). Bottom, migratory DCs (left) and lymphoid-resident DCs (right) in sLNs shown as the mean + SD (n=3-5 mice in 2-3 exp; Student's t-test). **(F)** Experimental design as in Figure 3F-G. Representative flow cytometry plots (left) and frequency of cells expressing CD45.1 or CD45.2 (right) (mean + SD; n=9 mice in 2 exp.). **(G)** Ears of B6 WT mice were painted with 0.5% TRITC. 48-96 hrs later, sLN were collected and processed for flow cytometry analysis. %TRITC^{+} cells shown as the mean + SD (n=4 mice in 2-3 exp.). chr: chromosome; gRNA: guide RNA; HA: homologous arm; bp: base pair; hrs: hours; Mig: Migratory; Res: lymphoid-resident.

Supplementary Figure 5

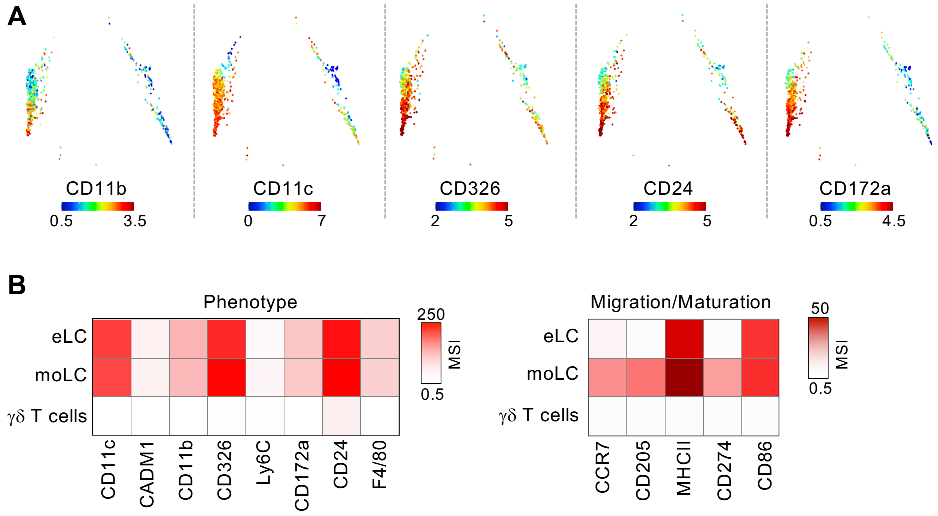

FIGURE S5. Epidermal moLCs express higher levels of migration/maturation markers. B6 *Cd207*^{DTR} mice were inoculated with 50ng/gr body weight DT or left untreated. Two months after DT inoculation, whole skin (A) or epidermis (B) cell suspensions were collected, and analyzed by CyTOF. **(A)** Expression of the indicated markers in skin LCs, shown in the PHATE map of Figure 4A. **(B)** Expression of the indicated markers in epidermal eLCs (-DT) and moLCs (+DT) (n=2 mice in 2 exp.). MSI: Mean Signal Intensity.

Supplementary Figure 6

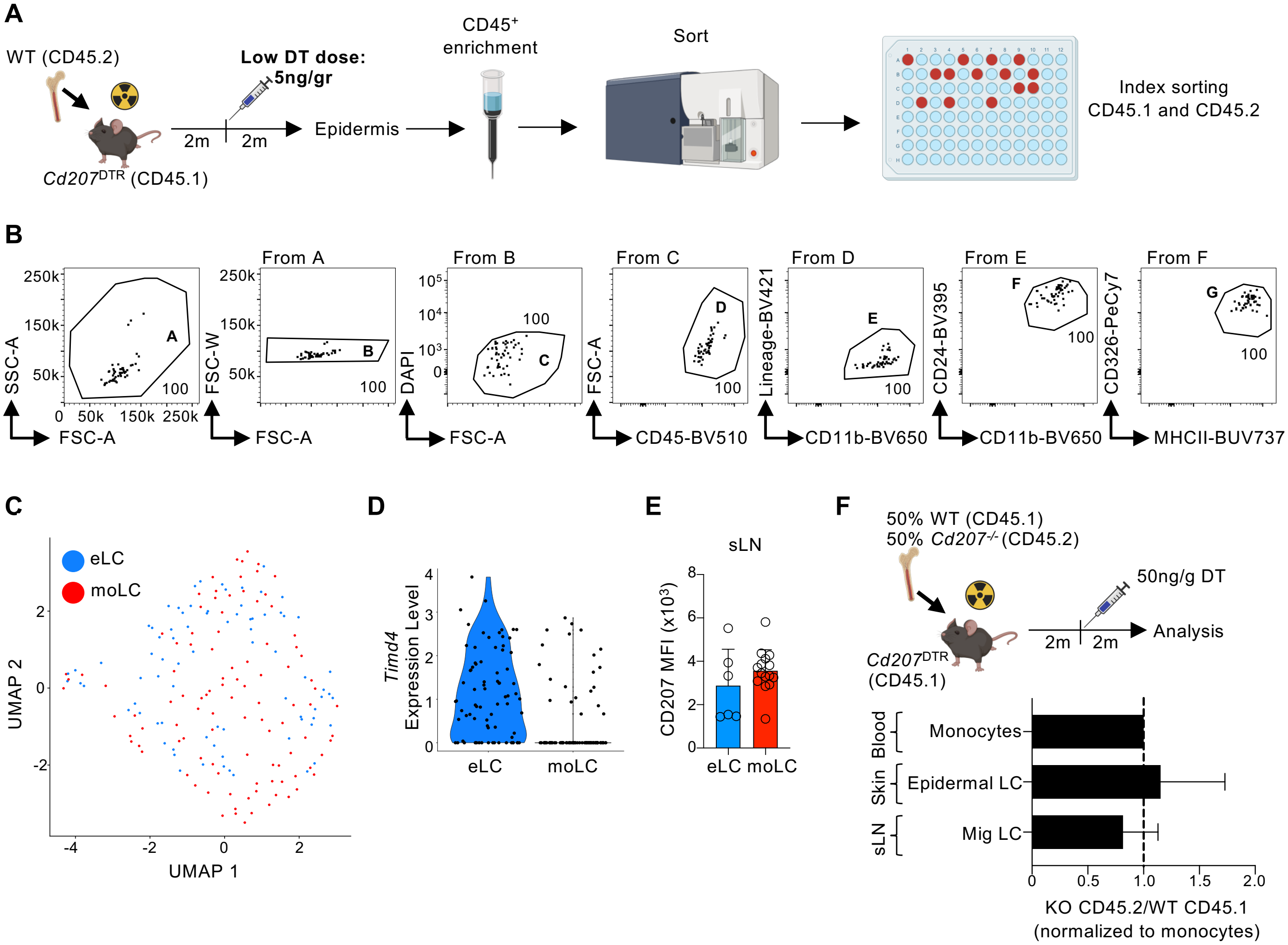

FIGURE S6. Transcriptomic analysis of epidermal eLCs and moLCs. (A-D) B6 CD45.1 *Cd207*^{DTR} mice were lethally irradiated and transplanted with CD45.2 WT bone marrow. Two months later, a low dose of 5 ng/gr body weight DT was inoculated. Epidermal eLCs (CD45.1) and moLCs (CD45.2) were analyzed by SMART-seq2 two months post-DT. **(A)** Schematic of the experimental design. **(B)** Epidermal LCs were sorted using Fig.S2A strategy (lineage markers included Ly6G and SiglecF). Shown is the cell purity after sort via index sorting. **(C)** UMAP of eLCs (CD45.1) and moLCs (CD45.2). **(D)** Violin plot of *Timd4* expression. **(E)** B6 *Cd207*^{DTR} were inoculated with 50 ng/gr body weight DT or left untreated. Two months later, sLN were collected and stained for flow cytometry. Expression of CD207 in migratory eLCs (-DT) and moLCs (+DT) in sLN, shown as the mean + SD (n=6-15 in 4 exp.; Student's t-test). **(F)** B6 *Cd207*^{DTR} CD45.1 mice were lethally irradiated and transplanted with 50% WT (CD45.1) and 50% *Cd207*$^{-/-}$ bone marrow (CD45.2). Two months later, 50ng/gr body weight DT was inoculated. Skin, sLN, and blood were analyzed two months post-DT. Top, schematic of the experimental design. Bottom, ratio CD45.2/CD45.1 positive cells normalized to monocytes, and shown as the mean + SD (n=4 in 2 exp.).

TABLE S1: Antibodies used for flow cytometry analysis and cell sorting.

Antibody	Source	Cat#	RRID
Anti-mouse B7-H1 (MIH5) SuperBright 780	eBioscience	78-5982-82	RRID: AB_2724081
Anti-mouse CCR7 (4B12) APC-eFluor®780	Thermo Fisher Scientific	47-1971-82	RRID:AB_2573974
Anti-mouse CD11b (M1/70) BV650	BioLegend	101239	RRID: AB_11125575
Anti-mouse CD11b (M1/70) APC-eFluor®780	Thermo Fisher Scientific	47-0112-82	RRID:AB_1603193
Anti-mouse CD11b (M1/70) eFluor®450	eBioscience	48-0112	RRID: AB_1582237
Anti-mouse CD11c (HL3) PE	BD Biosciences	561044	RRID:AB_2033996
Anti-mouse CD11c (HL3) BUV737	BD Biosciences	564986	RRID: AB_2870123
Anti-mouse CD172a (P84) BUV395	BD Biosciences	740282	RRID:AB_2740021
Anti-mouse CD19 (6D5) Alexa Fluor® 700	BioLegend	115528	RRID: AB_493735
Anti-mouse CD19 (eBioD3) eFluor®450	eBioscience	48-0193-82	RRID: AB_2734905
Anti-mouse CD207 (ebioL31) PE	Thermo Fisher Scientific	12-2075-82	RRID:AB_763452
Anti-mouse CD207 (L31) AF647	home-made		
Anti-mouse CD24 (M1/69) APC-eFluor®780	eBioscience	47-0242-82	RRID: AB_10853190
Anti-mouse CD24 (M1/69) BUV395	BD Biosciences	744471	RRID: AB_2742259
Anti-mouse CD25 (PC61) PerCP Cy5.5	BioLegend	102030	RRID: AB_893288
Anti-mouse CD3e (17A2) eFluor®450	BioLegend	100228	RRID:AB_2562553
Anti-mouse CD3e (17A2) Alexa Fluor® 700	BioLegend	100216	RRID: AB_493697
Anti-mouse CD326 (G8.8) PE	eBioscience	12-5791	RRID: AB_953615
Anti-mouse CD326 (G8.8) Alexa Fluor® 700	Biolegend	118239	RRID:AB_2810353
Anti-mouse CD326 (G8.8) PE-Cy7	eBioscience	25-5791-80	RRID: AB_1724047
Anti-mouse CD4 (GK1.5) BUV395	BD Biosciences	563790	RRID: AB_2738426
Anti-mouse CD45 (30-F11) Brilliant Violet 510™	BioLegend	103138	RRID: AB_2563061
Anti-mouse CD45 (30-F11) BV785	BioLegend	103149	RRID: AB_2564590
Anti-mouse CD45.1 (A20) PE	BD Biosciences	561872	RRID:AB_10897174
Anti-mouse CD45.1 (A20) APC-eFluor®780	Thermo Fisher Scientific	47-0453-82	RRID:AB_1582228
Anti-mouse CD45.2 (104) BV650	BioLegend	109835	RRID:AB_2563065
Anti-mouse CD45.2 (104) PE	BioLegend	109807	RRID:AB_313445
Anti-mouse CD45.2 (104) PE-Cy7	BioLegend	109829	RRID: AB_1186098
Anti-mouse CD64 (X54-5/7.1) APC	BioLegend	139306	RRID: AB_11219391
Anti-mouse CD86(GL1) AF647	Biolegend	105019	RRID:AB_493465
Anti-mouse F4/80 (BM8) eFluor®450	eBioscience	48-4801-82	RRID: AB_1548747
Anti-mouse Foxp3 (FJK-16s) APC	eBioscience	17-5773-82	RRID: AB_469457
Anti-mouse Ki67 (SolA15) PE	Thermo Fisher Scientific	12-5698-82	RRID:AB_11150954
Anti-mouse Ly6C (HK1.4) PerCP Cy5.5	Thermo Fisher Scientific	45-5932-82	RRID:AB_2723343
Anti-mouse Ly6C (HK1.4) BV510	Biolegend	128033	RRID:AB_2562351
Anti-mouse Ly6C (HK1.4) Alexa Fluor® 700	BioLegend	128024	RRID: AB_10643270
Anti-mouse Ly6G (1A8) BUV395	BD Biosciences	563978	RRID:AB_2716852
Anti-mouse Ly6G (1A8) Alexa Fluor® 700	BioLegend	127622	RRID: AB_10643269
Anti-mouse MHCII (M5/114.15.2) BUV737	BD Biosciences	748845	RRID:AB_2873248
Anti-mouse MHCII (M5/114.15.2) Brilliant Violet 510™	BioLegend	107635	RRID: AB_2561397
Anti-mouse NK1.1 (PK136) eFluor®450	Thermo Fisher Scientific	48-5941-82	RRID: AB_2043877
Anti-mouse NK1.1 (PK136) Alexa Fluor® 700	BioLegend	108730	RRID: AB_2291262
Anti-mouse SiglecF (E50-2440) eFluor®450	BD Biosciences	562681	RRID:AB_2722581
Anti-mouse Tim4 (RMT4-54) PE	Biolegend	130005	RRID:AB_2201843
Anti-mouse XCR1 (ZET) APC	BioLegend	148205	RRID:AB_2563931
Anti-mouse XCR1 (ZET) PerCP Cy5.5	BioLegend	148208	RRID: AB_2564364

TABLE S2: Antibodies used for CyTOF analysis.

Metal	Marker	Clone	Company/RRID
Pd104	CD45	30-F11	Thermo Fisher Scientific AB_467251
Pd106	CD45	30-F11	Thermo Fisher Scientific AB_467251
Pd108	CD45	30-F11	Thermo Fisher Scientific AB_467251
Y89	CD45	30-F11	Fluidigm RRID: AB_2651152
In113	CD45	30-F11	Thermo Fisher Scientific AB_467251
In115	PDCA1	eBio927	Biolegend RRID: AB_1953281
Pr141	Ly6G	1A8	Fluidigm RRID: AB_2814678
Nd143	CD11b	M1/70	Fluidigm RRID: AB_2811240
Nd144	B220	RA3-6B2	Fluidigm RRID: AB_2811239
Nd145	MHCII	M5/114.15.2	Biolegend RRID: AB_2563771
Nd146	EpCAM	G8.8	Biolegend RRID: AB_2563743
Sm147	CD172a	P84	Biolegend RRID: AB_11203723
Nd148	CD127	A7R34	Biolegend RRID: AB_2563716
Sm149	CD19	6D5	Fluidigm RRID: AB_2814679
Nd150	Ly6C	HK1.4	Biolegend RRID: AB_2563783
Eu151	CD64	X54-5/7.1	Fluidigm RRID: AB_2814680
Sm152	CD3	1452C11	RRID:AB_468847
Eu153	CD335	29A1.4	Biolegend RRID: AB_2563744
Sm154	CD24	M1/69	Biolegend RRID: AB_2563732
Gd155	FceR1a	Mar-1	Biolegend RRID: AB_2563768
Gd156	CD14	Sa14-2	Fluidigm RRID: AB_2814681
Gd157	CD205	NLDC	homemade
Gd158	SiglecH	551	Biolegend RRID: AB_1227757
Tb159	F4/80	BM8	Fluidigm RRID: AB_2811238
Gd160	MGL2	URA-1	Biolegend RRID: AB_2562711
Dy161	CD8	53-6.7	Biolegend RRID: AB_2562796
Bi209	CD11c	N418	Fluidigm RRID: AB_2814682
Dy163	XCR1	Zet	Biolegend RRID: AB_2563841
Dy164	CCR7	4B12	Fluidigm RRID: AB_2814683
Ho165	CCR2	475301	R&D Systems RRID: AB_10718414
Er166	Cx3CR1	SA011F11	Biolegend RRID: AB_2564313
Er167	CD115	AFS98	Biolegend RRID: AB_2563709
Er168	CD116	698423	ThermoFisher Scientific RRID AB_2608189
Tm169	PDL1	MiH5	Thermo Fisher Scientific RRID: AB_467781
Yb171	CD103	M290	BD Pharmingen RRID: AB_394995
Yb172	CD86	GL1	Biolegend RRID: AB_313144
Yb173	CD117	2B8	Fluidigm RRID: AB_2811230
Yb174	CD135	A2F10	ThermoFisher Scientific RRID AB_467481
Lu175	Cadm1	3E1	MBL RRID: AB_592783
CD116	Ly6D	49-H4	BD Pharmingen RRID: AB_396664

TABLE S3: Antibodies used for microscopy analysis.

Antibody	Fluorohrome	RRID	Isotype	Final concentration	Source	2nd antibody	RRID 2nd antibody	Final concentration 2nd antibody	Source 2nd antibody
CD45.1	Biotin	AB_395042	Mouse (SJL) IgG2a, κ	3 μg/ml	BD Biosciences	Sva Brilliant Violet 421	-	2 μg/ml	Biolegend
CD45.2	Purified	AB_467261	Mouse (SJL) IgG2a, κ	3 μg/ml	Thermo Fisher Scientific	anti-Rat Alexa Fluor® 555	AB_2896336	1 μg/ml	Thermo Fisher Scientific
GFP	Purified	AB_221569	Rabbit IgG	4 μg/ml	Thermo Fisher Scientific	anti-Rabbit Alexa Fluor® 488	AB_143165	1 μg/ml	Thermo Fisher Scientific
I-A/I-E	Alexa Fluor® 647	AB_493525	Rat IgG2b, κ	1 μg/ml	Biolegend	-	-	-	-
TCRγδ	Alexa Fluor® 488	AB_2562771	Ar. Hamster IgG	2.5 μg/ml	Biolegend	-	-	-	-
TCRγδ	PE	AB_465934	Ar. Hamster IgG	1 μg/ml	Thermo Fisher Scientific	Anti-Ar. Hamster Alexa Fluor® 555	AB_2925787	1 μg/ml	Thermo Fisher Scientific
